# Epigenetic reprogramming of epithelial mesenchymal transition in triple negative breast cancer cells with DNA methyltransferase and histone deacetylase inhibitors

**DOI:** 10.1186/s13046-018-0988-8

**Published:** 2018-12-14

**Authors:** Yanrong Su, Nathan R. Hopfinger, Theresa D. Nguyen, Thomas J. Pogash, Julia Santucci-Pereira, Jose Russo

**Affiliations:** The Irma H. Russo, MD Breast Cancer Research Laboratory, Fox Chase Cancer Center-Temple University Health System, Philadelphia, PA 19111 USA

**Keywords:** Triple negative breast cancer, Epithelial mesenchymal transition, DNA methyltransferase, Histone deacetylase

## Abstract

**Background:**

Triple negative breast cancer (TNBC) is an aggressive neoplasia with no effective therapy. Our laboratory has developed a unique TNBC cell model presenting epithelial mesenchymal transition (EMT) a process known to be important for tumor progression and metastasis. There is increasing evidence showing that epigenetic mechanisms are involved in the activation of EMT. The objective of this study is to epigenetically reverse the process of EMT in TNBC by using DNA methyltransferase inhibitors (DNMTi) and histone deacetylase inhibitors (HDACi).

**Methods:**

We evaluated the antitumor effect of three DNMTi and six HDACi using our TNBC cell model by MTT assay, migration and invasion assay, three dimensional culture, and colony formation assay. We then performed the combined treatment both in vitro and in vivo using the most potent DNMTi and HDACi, and tested the combined treatment in a panel of breast cancer cell lines. We investigated changes of EMT markers and potential signaling pathways associated with the antitumor effects.

**Results:**

We showed that DNMTi and HDACi can reprogram highly aggressive TNBC cells that have undergone EMT to a less aggressive phenotype. SGI-110 and MS275 are superior to other seven compounds being tested. The combination of SGI with MS275 exerts a greater effect than single agent alone in inhibiting cell proliferation, motility, colony formation, and stemness of cancer cells. We also demonstrated that MS275 and the combination of SGI with MS275 exert in vivo antitumor effect. We revealed that the combined treatment synergistically reverses EMT through inhibiting EpCAM cleavage and WNT signaling, suppressing mutant p53, ZEB1, and EZH2, and inducing E-cadherin, apoptosis, as well as histone H3 tri-methylation.

**Conclusions:**

Our study showed that DNMTi and HDACi exert antitumor activity in TNBC cells partially by epigenetically reprograming EMT. Our findings strongly suggest that TNBC is sensitive to epigenetic therapies. Therefore, we propose a new strategy to treat TNBC by using the combination of SGI-110 with MS275, which exerts superior antitumor effects by simultaneously targeting multiple pathways.

**Electronic supplementary material:**

The online version of this article (10.1186/s13046-018-0988-8) contains supplementary material, which is available to authorized users.

## Background

Triple negative breast cancer (TNBC) is a subtype of breast cancer negative for hormone receptors and human epidermal growth factor receptor 2. Although TNBC only accounts for 15–20% of breast cancers [1], the mortality for women with TNBC is substantially higher than those women with other subtypes of tumors, mostly due to the development of metastases [2]. Compared to other breast cancer subtypes, TNBC is more aggressive, and more difficult to treat. Radiotherapy and chemotherapy are the two main therapies combined with surgery for TNBC. Standard chemotherapies show some effects for subtypes of TNBC [3], however, patients with advanced disease typically respond poorly to current chemotherapies [4]. The targeted therapies are an urgent need for this disease considering its disproportionate contribution to breast cancer death [5].

Epithelial-mesenchymal transition (EMT) is a process by which epithelial cells lose their epithelial characteristics and acquire mesenchymal phenotype. The crucial steps of tumor metastasis are thought to be enabled by EMT [6, 7]. EMT is also associated with cancer stem cell (CSC) properties [8]. Breast CSC with CD44^+^CD24^−/low^ phenotype can be generated through EMT [9, 10]. TNBC is a heterogeneous group of cancers, intrinsic subtype analysis showed approximately 80% of TNBC are basal-like and claudin-low tumors. Similarly, majority of TNBC cell lines are also assigned to basal-like and mesenchymal-like subtype [11]. Expression of EMT markers and CD44^+^CD24^−/low^ are significantly higher in basal-like and mesenchymal-like subtype TNBC, and are associated with poor outcome [12, 13]. Therefore, targeting EMT is of great interest for developing novel therapeutics for TNBC.

Increasing evidence showed that epigenetic mechanisms are involved in the activation of EMT process. The well-studied event is the epigenetic regulation of E-cadherin expression. The *CDH1* promoter hyper-methylation is a part of entire EMT program resulting breast tumor cells with a more aggressive phenotype [14]. In addition, E-cadherin expression is also repressed by a number of EMT inducers including SNAIL, SLUG, ZEB1, ZEB2, and TWIST [15–18]. The repression of E-cadherin by these repressors are associated with histone deacetylase (HDAC) [16, 19–22] .

The reversibility of epigenetic alterations and the importance of DNA methylation and histone acetylation in tumor progression have resulted in the development of pharmacologic inhibitors for epigenetic therapy. In this study, we determined whether DNA methyltransferase inhibitor (DNMTi) and histone deacetylase inhibitor (HDACi) have antitumor effect on TNBC cells by reprograming EMT. We took advantage of the TNBC cell model established in our lab, which consists of the normal like human breast epithelial cell line MCF10F, the cell line trMCF which was transformed from MCF10F, and the tumorigenic cell line bsMCF derived from trMCF, as well as two highly tumorigenic and metastatic cancer cell lines XtMCF and LmMCF developed from bsMCF [23]. The bsMCF, XtMCF, and LmMCF cells have undergone EMT, displaying mesenchymal phenotype [10]. The advantage of this unique cell model is that all the cells are derived from the same genotype present in the MCF10F cells and we have been able to identify epigenetic and genomic changes during the process of neoplastic transformation. Using this cell model, we first evaluated the antitumor efficacy of a panel of DNMTi and HDACi by different assays, and then performed the combined treatment using the selected compounds. We investigated the changes of EMT markers and potential signaling pathways associated with the antitumor effect. The results described here strongly support the notion that malignant phenotype of TNBC cells can be suppressed by epigenetic reprogramming of EMT.

## Methods

### Cell lines and compounds

Triple negative human breast epithelial/cancer cell lines MCF10A, MCF10F, trMCF, bsMCF, bsMCF-luciferase (bsMCF-luc), XtMCF, and LmMCF cell lines were cultured as described in the previous studies [10, 23]. Other breast cancer cell lines used in the study are: MCF7, T47D, BT474, SK-BR-3, BT-549, MDA-MB-468, MDA-MB-231, Hs578t, HCC1954, Sum149pt, and Sum159pt. The media used for these cells are described in Additional file 1. The subtype information of these cell lines are shown in Additional file 2: Table S1. All cell lines used for this study were authenticated or certified, and used in less than ten passages after recovery.

Three DNA methyltransferase inhibitors (DNMTi): 5-Azacytidine (AZA), Decitabine (DAC), and Guadecitabine/SGI-110 (SGI), and six histone deacetylase inhibitors (HDACi): JNJ-26481585 (JNJ), Vorinostat (SAHA), Entinostat (MS275), SB939, LBH589, and Tubastatin A HCL (Tub), were used to treat cells. Two forms of SGI-110 were used, SGI-110 powder for injection was used for RTCA assay and in vivo study, and SGI-110 salt was used for all other in vitro studies. The information and preparation of these drugs are shown in Additional file 3: Table S2.

### Drug treatment and MTT cell proliferation assay

Cells were plated in 96-well plates for MTT assay (see Additional file 1), or in plastic flasks or dishes for in vitro phenotypic studies. Cells were plated on day 1, treated with drug at a dose around IC50 on day 2, cultured for additional 96 h; then cells were trypsinized, examined the viability by trypan blue staining, and the viable cells were used for phenotypic studies unless specified.

### Wound healing assay and Matrigel invasion assay

Viable cells after treatment with the dose around IC50 were used for wound healing assay as previously described [10] and Matrigel invasion assay (see Additional file 1). Wound healing assay was presented as percent wound closed, and invasion assay was presented as percent of control.

### RTCA migration and invasion assay

Cell migration and invasion were also measured by RTCA (real-time cell analyzer) assay using xCELLigence system (ACEA Biosciences INC, San Diego, CA). Cells were treated with the dose around IC50 (refers to high dose) or a dose that inhibits cell growth by around 20% (refers to low dose), 96 h after treatment, cells were trypsinized, and the viable cells were seeded in RTCA plate for the assay (Additional file 1). As at the late time point, the impedance microelectrodes of the RTCA plate not only measure cell migration and invasion from the upper chamber to the lower chamber but also the cell proliferation on the underside of the membrane, we chose a time point in the slope of the cell index curves based on cell doubling time (20 to 30 h for these cell lines) to present data by bar graphs.

### Three dimensional (3D) culture in collagen matrix

3D culture was done as previously described [10]. Drug-treated or control cells were used for 3D culture. At the end of the examination period, images were acquired, the number of ductal structures or masses formed in the collagen was quantified and classified.

### Colony formation in agar methylcellulose matrix

Drug-treated or control cells were used for colony formation assay as described [10]. The colony formation was monitored under microscope. At the end of the examination period, cells were stained and the images were acquired. The number and the size of colonies were quantified.

### Immunofluorescence (IF)

Immunofluorescence staining was performed using antibodies vimentin, EpCAM{Abbiotech}, EpCAM(VU1D9), EpCAM(E144), or cleaved caspase 3. The information of the antibodies is shown in Additional file 4: Table S3. The details of staining are described in Additional file 1.

### Western blotting (WB)

Primary antibodies E-cadherin, vimentin, SLUG, EpCAM(VU1D9), TCF4, p53, EZH2, ZEB1, H3K27me3, Histone H3, Beta actin, and GAPDH were used for WB (Additional file 1). The information of the antibodies is shown in Additional file 4: Table S3.

### Tumorsphere formation assay

Single cell suspension from drug- or vehicle-treated cells was plated in ultra-low attachment 6-well plate at a density of 50,000 cells/well in culture media supplemented with 10 ng/ml EGF, 20 ng/ml bFGF, and 1 × B27. Four days after culture, images of tumorspheres were acquired using NIKON ECLIPSE TS100 microscope. The number of spheres were counted and graphed.

### In vivo animal studies

Animal studies were conducted in the laboratory animal facility at Fox Chase Cancer Center (FCCC) using protocol approved by the IACUC committee. Eight to nine weeks old female CB17/SCID or NOD/SCID mice from FCCC animal facility were used. 5 × 10^4^ viable XtMCF or LmMCF cells in 100 μl of PBS were mixed with 100 μl of Matrigel (BD Biosciences) and injected subcutaneously to the inguinal mammary fat pad. The treatment for CB17/SCID mice started from three days post cell injection, mice were randomly divided into four groups with 10 mice per group: (1) vehicle control; (2) SGI (1.5 mg/kg, Monday and Wednesday for XtMCF model; Monday, Wednesday, and Friday for LmMCF model; s.c. injection); (3) MS275 (0.5 mg/kg, Thursday; i.p. injection); (4) SGI + MS275. The treatment for NOD/SCID mice started from two weeks post cell injection when a xenograft was palpable. Mice were assigned into four groups: (1) vehicle control; (2) SGI (2.0 mg/kg, Monday, Wednesday, Friday; s.c. injection; two weeks); (3) MS275 (1.0 mg/kg, Thursday; i.p.; three weeks); (4) SGI + MS275. Tumor size was measured with caliper and calculated using the formula ½*length*width^2^ twice a week.

The lung metastatic study was conducted by injecting 8 × 10^4^ viable LmMCF cells into the tail vein. Six days post cell injection, mice were randomly divided into two groups: (1) control; (2) MS275 (0.5 mg/kg, Thursday; i.p. injeciton). Mice were treated for three weeks, sacrificed 25 days post cell injection. Lungs were fixed in Bouin’s solution to visualize the metastases, and then processed and embedded in paraffin for histology examination.

### Immunohistochemical (IHC) staining

Paraffin-embedded mouse lung sections at 4 μm thickness were stained with anti-human vimentin antibody (BioGenex, Fremont, CA) to evaluate lung metastases. Staining was performed following the standard protocol using i6000 Autostainer (BioGenex). Super Sensitive™ Polymer-HRP Detection System (BioGenex) was used to detect the immunostaining.

### Statistical analysis

Statistical analysis was carried out using SigmaPlot 12.0 software (Systat Software Inc., San Jose, CA). Studies involving more than two groups were analyzed by one-way analysis of variance (ANOVA) followed by Tukey’s post-hoc multiple comparison tests or Dunn’s methods. Chi-square analysis was used to evaluate the distribution of masses formed in 3D culture in collagen. *P*-value less than 0.05 were considered statistically significant.

## Results

DNMTi and HDACi inhibit the growth of triple negative breast epithelial/cancer cells.

**Fig. 1:**
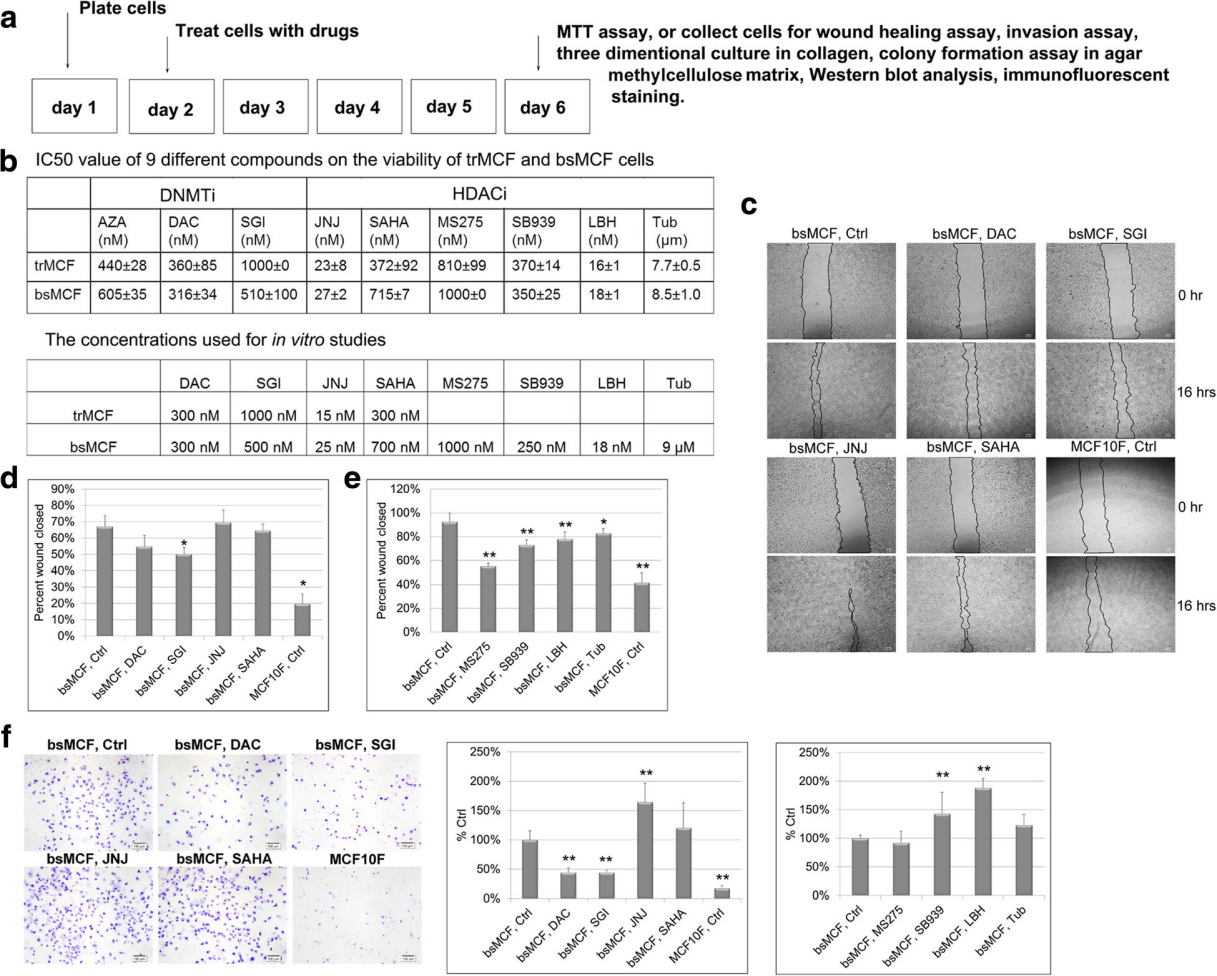
DNMTi and HDACi treatment inhibit proliferation, migration, and invasion of TNBC cells. **a** Schematic representation of the cell treatment for in vitro phenotypic studies. **b** Tables show the IC50 of the compounds and the concentrations used for in vitro assays. **c** Representative images of wound healing assay. Magnification, 40×. Scale bar, 60 μm. **d**-**e** Quantification of wound healing assay. **f** Representative images of Matrigel invasion assay and quantification of the invaded cells. Scale bar, 100 μm. (40×). *indicates *p* < 0.05, **indicates *p* < 0.01 compared to control. All experiments were repeated at least twice and one representative experiment is shown in the figure. This also applies to the experiments shown in other figures

To assess the effect of DNMTi and HDACi on the growth of triple negative breast epithelial/cancer cells, the transformed human breast epithelial cell line trMCF and mesenchymal-like cell line bsMCF [23], were treated as shown in Fig. 1a. The growth-inhibitory activity was assessed by MTT assay. All compounds induced a dose-dependent inhibition of proliferation, with the most sensitive to LBH and the least sensitive to Tub (Fig. 1b). The representative growth curves were shown in Additional file 5: Figure S1. The sensitivity of bsMCF cells to the three DNMTi was comparable. For comparison, both DAC and SGI were chosen for further studies. By contrast, the growth inhibitory effect of six HDACi varied a lot, therefore all six HDACi were used for further studies. The dose around IC_50_ was used to do the treatment for the following in vitro studies unless specified (Fig. 1b).

### DNMTi and HDACi inhibit bsMCF cell migration

To determine the effect of DNMTi and HDACi on cell migration, wound healing assay was performed. As shown in Fig. 1c and d, SGI but not DAC significantly inhibited migration of bsMCF cells (Wound closed: 66.9% ± 6.95% for control vs. 50.1% ± 4.2% for SGI over 16 h). Four out of six HDACi significantly inhibited bsMCF cells migration. Among them, MS275 showed the greatest inhibition (Wound closed: 92.3% ± 7.6% for control vs. 55.2% ± 2.6% for MS275, 72.9% ± 4.5% for SB939, 77.9% ± 5.8% for LBH, 82.9% ± 3.7% for Tub over 18 h) (Fig. 1d, e).

### DNMTi and HDACi differentially affect bsMCF cell invasion

We further examined whether the treatment affects cell invasion. Both DAC and SGI significantly suppressed bsMCF cell invasion by 56%. SAHA, MS275, and Tub did not have much effect. Interestingly, although the growth of bsMCF cells was significantly inhibited by JNJ, SB939, and LBH at very low doses treatment, the three drugs significantly increased cell invasion by 64% (JNJ), 43% (SB939), and 88% (LBH) (Fig. 1f), indicating that the inhibition of cell growth should not be used as the only parameter for selecting the drug for cancer treatment. Drugs like JNJ, SB939, and LBH can promote cell invasion while inhibiting cell growth, thus may accelerate the progression of the disease.

### DNMTi and HDACi reverse the behavior of bsMCF cells grown in collagen

**Fig. 2:**
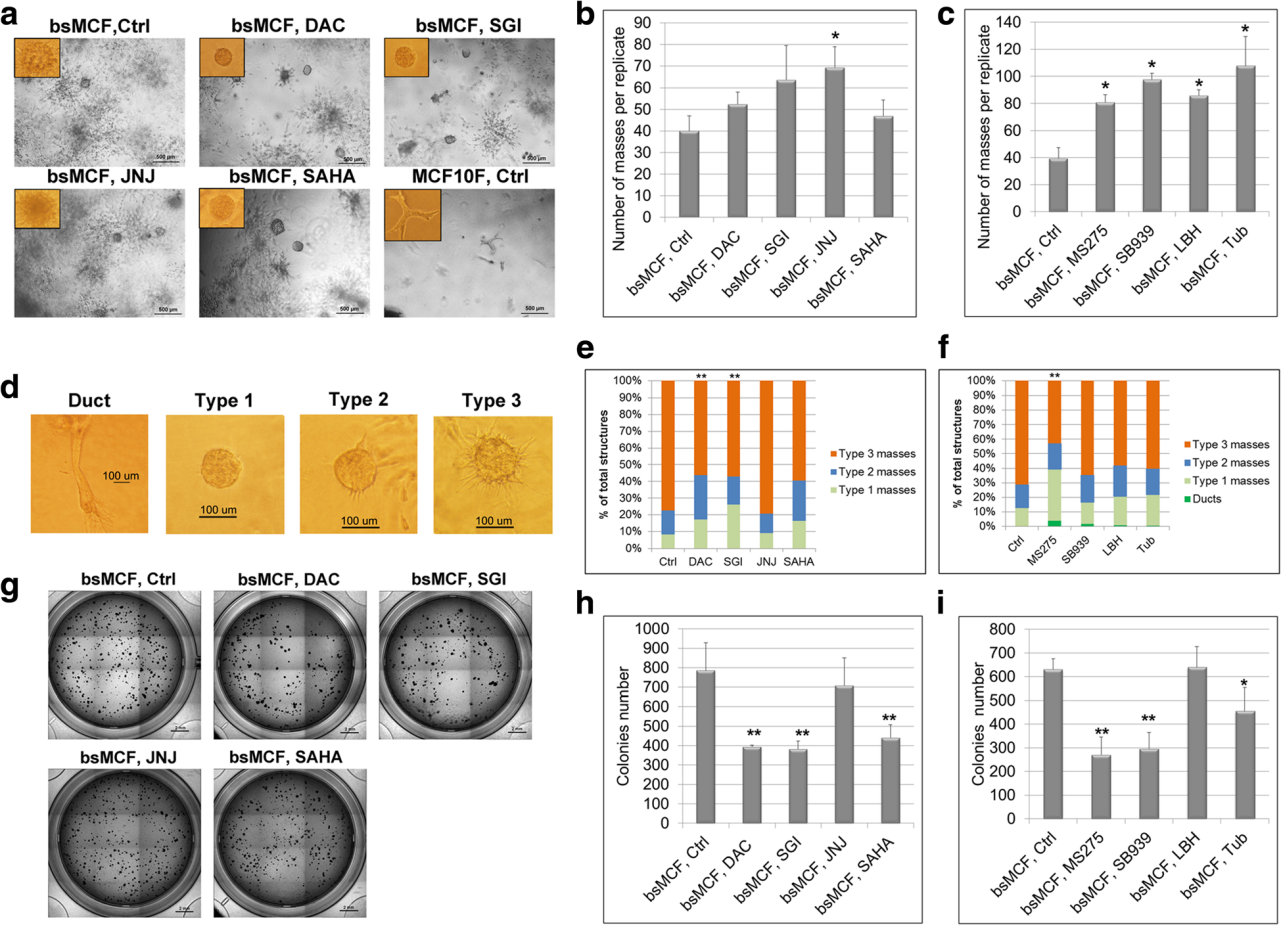
DNMTi and HDACi reverse bsMCF cells malignant behavior in collagen and inhibit colony formation. **a** Representative images of 3D culture in bovine type I collagen. Images of structures formed in collagen were acquired after 6 days of culture. Magnification, 40×. Scale bar, 500 μm. **b**-**c** Quantification of the number of masses formed in collagen. **d** Classification of the masses. Type 1 masses show smooth outer surface and no protrusions present; type 2 masses show rough outer surface with 1–5 protrusions; type 3 masses show large amount of protrusions (> 5), indicate more invasive phenotype. Magnification, 40× for duct, and 100× for type 1–3 masses. **e**-**f** Quantification of the types of the masses. DAC, SGI, and MS275 treatment increase the number of masses with fewer protrusions. **g** Representative images of colony formation in agar methylcellulose matrix. Images were acquired after 9 days of culture. Magnification, 2×. Scale bar, 2 mm. **h**-**i** Quantification of the number of the colonies formed in methylcellulose matrix. *indicates *p* < 0.05. ** indicates *p* < 0.01 compared to control

The 3D cell culture is a technique that more closely resembles the cell growth behavior in vivo. When cultured in 3D collagen matrix, MCF10F cells form branching ductal structures. Tumorigenic bsMCF cell line usually does not form ductal structures; instead, it forms solid mass displaying protrusions and shows disseminated growth of individual cells [10]. The number of solid masses may indicate the potential of tumor cells to form a structure mimicking the primary tumor with cell-cell contact, whereas the protrusions resemble cell invasion, and the disseminated cell growth exhibits the mesenchymal feature of the tumor cells. Treatment of JNJ, MS275, SB939, LBH, and Tub significantly increased the number of masses (Fig. 2a-c), suggesting the treatment increases cell-cell contact. The masses were then classified based upon the display of protrusions. DAC, SGI, and MS275 significantly increased type 1 masses, which showed no protrusions on the outer surface of the mass (Fig. 2d-f). SGI is superior to DAC, and MS275 is superior to other HDACi with regarding to the formation of type 1 masses, implying cells treated with SGI or MS275 are less invasive. It is noteworthy that DAC, SGI, and MS275 treatment reduced the disseminated growth of the individual cells (Fig. 2a and Additional file 6: Figure S2a), suggesting the reversal of mesenchymal feature by these treatments.

### DNMTi and HDACi inhibit anchorage independent growth of bsMCF cells

Anchorage independent growth of cells is usually correlated to the tumorigenicity of transformed cells. We next assessed the effect of drug treatment on the colony formation ability of bsMCF cells in methylcellulose matrix. DAC, SGI, SAHA, MS275, SB939, and Tub significantly reduced the colonies number formed by bsMCF cells. DAC and SGI were comparable in the extent of inhibition. MS275 was the most potent one among six HDACi in decreasing the colonies number (Fig. 2g-i; Additional file 6: Figure S2b).

### Combined treatments of DNMTi with HDACi are more effective in bsMCF cells

**Fig. 3:**
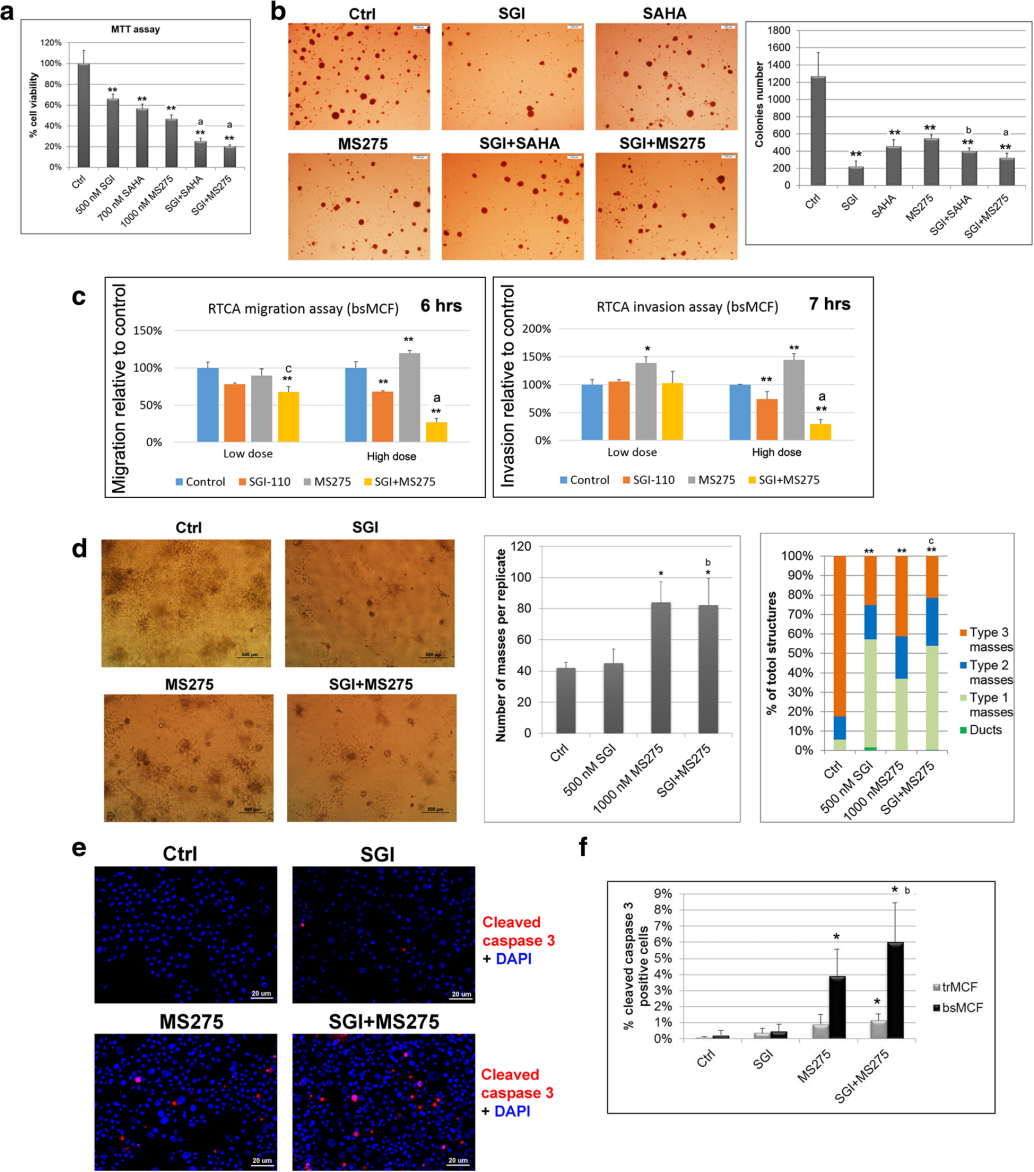
Combined treatment of DNMTi with HDACi is more effective in bsMCF cells. **a** Combined treatment of SGI with SAHA or MS275 induces greater inhibition in cell proliferation. **b** Colony formation in agar methylcellulose and quantification of colonies number. Magnification, 20×. Scale bar, 200 μm. **c** Quantification graphs at the time point of 6 h for cell migration, and 7 h for invasion by RTCA assay. Cells were first treated and then viable cells were used for both migration and invasion assay. **d** Representative images of 3D culture in collagen, and quantification of the number and types of masses formed by bsMCF cells. Magnification, 40×. Scale bar, 500 μm. **e** Representative images of cell apoptosis by immunofluorescence. bsMCF cells were treated with indicated agents, four days later, both floating and adherent cells were collected and cytospun on glass slides. Cells were fixed and stained with cleaved caspase 3 antibody. Magnification, 200×. Scale bar, 20 μm. **f** Quantification of cell apoptosis. The symbols used for indicating statistical results are as follows: * indicates *p* < 0.05 when compared to control. ** indicates *p* < 0.01 when compared to control. a indicates *p* < 0.01 when compared the combined treatment with single agent. b indicates *p* < 0.05 when compared the combined treatment with SGI. c indicates *p* < 0.05 when compared the combined treatment with MS275. This rule applies to other figures too

The treatment of DNMTi in combination with HDACi in cancers has been shown more effective than the treatment of single agent in some studies [24, 25]. We evaluated the effect of combined treatment using bsMCF cells. Treatment of SGI combined with SAHA (SGI + SAHA) or with MS275 (SGI + MS275) induced a greater inhibition in cell proliferation compared to single agent (Fig. 3a). In addition, colony formation assay also showed SGI + SAHA and SGI + MS275 formed fewer colonies compared to SAHA or MS275 treatment alone (Fig. 3b). SGI + MS275 is better than SGI + SAHA in inhibiting both cell proliferation and colony formation, thus SGI + MS275 was chosen for further study. The effect of the combined treatment on cell migration and invasion was evaluated by RTCA assay at two different doses (Additional file 7: Figure S3). A significant decrease of cell migration was observed by SGI + MS275 but not by single agent at low dose treatment. At high dose, SGI + MS275 induced a significant decrease in both migration and invasion compared to control or single agent (Fig. 3c).

When examined cell growth in collagen matrix, the treatment of SGI + MS275 formed more masses than SGI treatment alone, although there was no difference in the type of masses between these two groups. By contrast, there was no significant difference in the number of masses between the SGI + MS275 and MS275 treatment, whereas SGI + MS275 increased the proportion of masses with smooth outer surface than MS275 treatment alone (Fig. 3d), indicating the combined treatment is better than single agent in certain characteristics.

As floating cells were observed when treated with MS275 or SGI + MS275, cell apoptosis was evaluated by staining with cleaved caspase 3 on cytospun slides, The result showed SGI + MS275 induced significant apoptosis than single agent. In addition, the apoptosis was more pronounced in bsMCF cells than in trMCF cells (Fig. 3e, f).

### SGI combined with MS275 inhibits cell growth, colony formation, and stemness of two highly tumorigenic and metastatic cell lines XtMCF and LmMCF

**Fig. 4:**
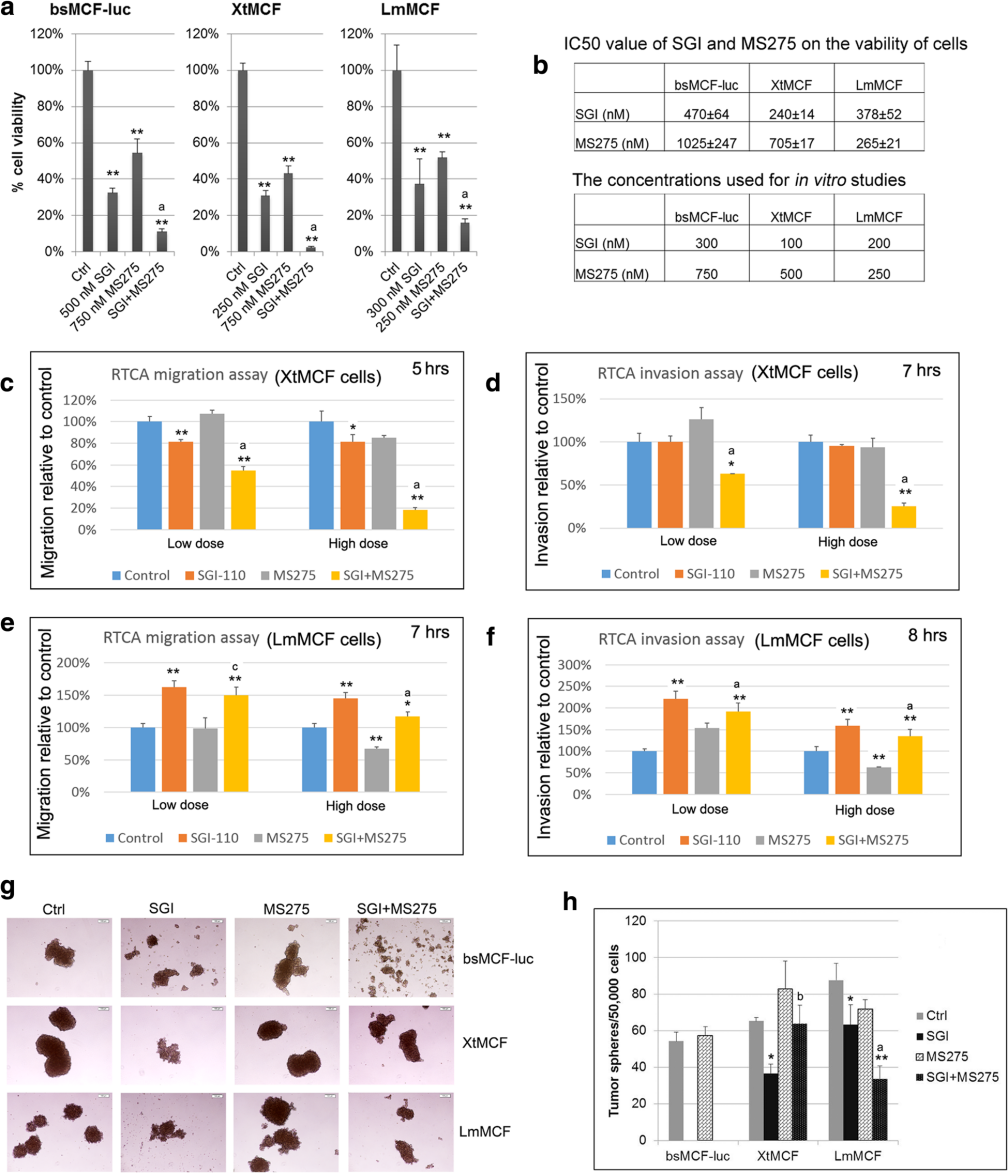
Effects of SGI, MS275, or the combined treatment in bsMCF-luc, XtMCF, and LmMCF cell lines. **a** Combined treatment of SGI with MS275 significantly inhibits cell growth when compared with the single agent examined by MTT assay. **b** Tables show the IC50 of SGI and MS275, and the concentrations used for in vitro studies. **c**, **e** Quantification graphs for RTCA cell migration assay. **d**, **f** Quantification graphs for RTCA cell invasion assay. **g** Representative images of tumorspheres. Magnification, 40×. Scale bar, 100 μm. **h** Quantification of tumorshperes number. The tumorspheres formed by bsMCF-luc cells after the treatment with SGI or the combination were dissociated automatically and not counted

The above finding prompted us to investigate whether these treatments have in vivo antitumor activity. For this purpose, we decided to use two highly tumorigenic and metastatic TNBC cell lines XtMCF and LmMCF [10]. We first evaluated the in vitro antitumor activity. Compared to parental bsMCF-luc cells, the proliferation of XtMCF and LmMCF cells was more sensitive to SGI or MS275 (Additional file 8: Figure S4). The combined treatment significantly reduced cell viability compared to single agent (Fig. 4a). Concentrations lower than IC50, as indicated in Fig. 4b, were then used for in vitro phenotypic studies. The colony formation assay (Additional file 9: Figure S5) showed that SGI and SGI + MS275 induced a marked decrease of colony number in all three cell lines. Whereas MS275 decreased colony number in bsMCF-luc and XtMCF cells only. SGI + MS275 reduced colony number compared to MS275 alone in bsMCF-luc and LmMCF cell lines.

RTCA assay was used to evaluate the effect of the treatment on migration and invasion. The doses and the cell index plots were shown in Additional file 10: Figure S6. For XtMCF cells, SGI treatment significantly inhibited cell migration (Fig. 4c) but not invasion. MS275 did not have much effect at any dose tested. Strikingly, SGI + MS275 significantly inhibited both migration and invasion at both low and high dose (Fig. 4c, d). LmMCF are not invasive without adding additional growth factors in in vitro study. With basic FGF as a chemoattractant, only high dose MS275 showed inhibition in cell migration and invasion for LmMCF cell line, whereas SGI-110 or the SGI + MS275 promoted both migration and invasion at low or high dose, one explanation could be the treatments changed the expression of the growth factor receptor and that might have influence on migration or invasion (Fig. 4e, f).

We observed bsMCF-luc, XtMCF, and LmMCF cells have undergone EMT and are able to generate tumorspheres [10]. Tumorsphere formation assay showed SGI treated cells formed tumorspheres at the beginning in all three cell lines, however, the tumorspheres started to dissociate after four days culture, this phenomenon was more striking in bsMCF-luc cell line than in other two cell lines. Treatment of MS275 alone did not significantly affect the formation of tumorspheres. SGI + MS275 resulted in dissociation of almost all tumorspheres formed by bsMCF-luc cells, reduced mammospheres formed by XtMCF compared to MS275 alone, and significantly reduced the number of tumorspheres formed by LmMCF cells compared to single agent (Fig. 4g, h).

The above results show that the combined treatment was better than single agent in majority of the parameters evaluated, while for the effects on certain aspects such as colony formation or cancer stemness, the combined treatment was better than one of the two agents only in certain cell lines, suggesting that different pathways might be involved in different cell lines, and patient selection is very important for a treatment to exert its best effect in the clinics.

### MS275 alone, and SGI combined with MS275 inhibit xenograft growth of XtMCF cells; MS275 reduces lung metastases of LmMCF cells in vivo

**Fig. 5:**
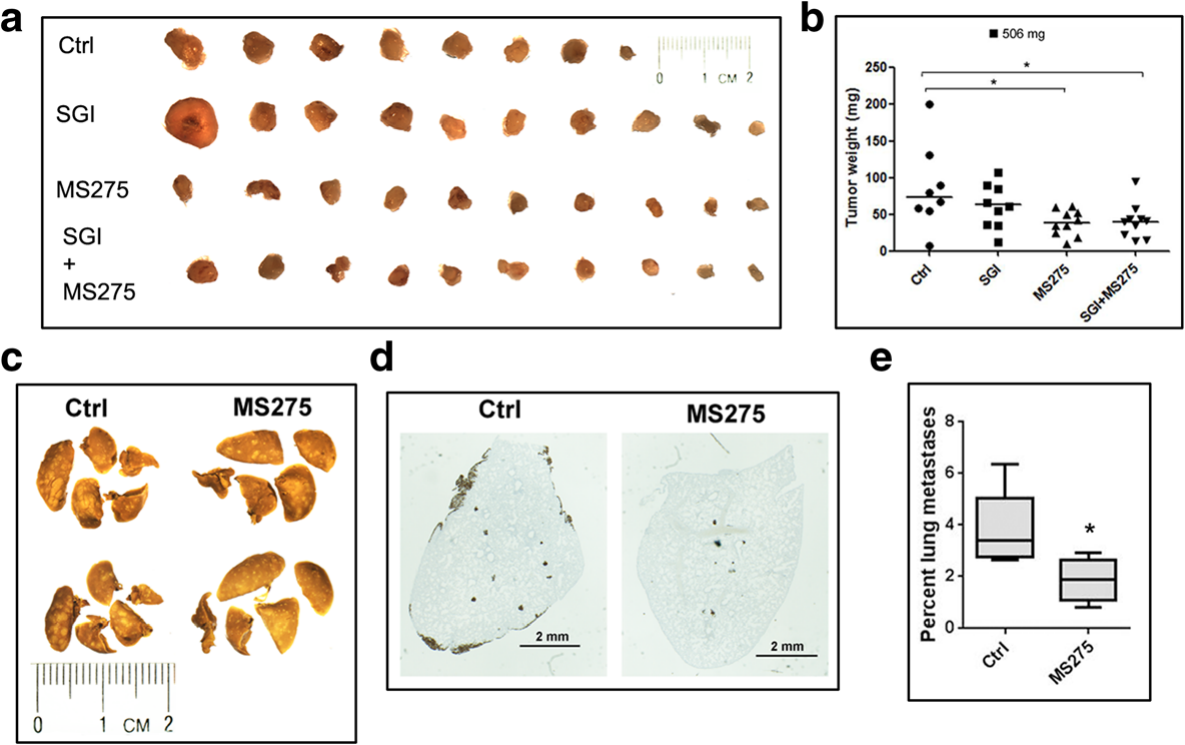
MS275, or the combination of SGI with MS275 suppress TNBC growth or metastasis. **a** 5 × 10^4^ XtMCF cells were injected to the mammary fat pad of 8–9 weeks old female CB17/SCID mice. Three days after cells injection, mice were treated with indicted compounds for three weeks. Mice were sacrificed 31 days post cells injection. Images of XtMCF xenografts are shown. Magnification, 6.3×. **b** Dot plot shows the tumor weight at sacrifice, the line in each group indicates the median value. **c** 8 × 10^4^ LmMCF cells were injected into the tail vein of 8–9 weeks old female CB17/SCID mice. Six days after cells injection, mice were treated with vehicle control or MS275 once a week for three weeks. Mice were sacrificed 25 days post cells injection. Representative images of lungs fixed with Bouin’s solution show lung metastases on lung surface. Magnification, 8×. **d** Immunohistochemical staining to vimentin on lung sections show metastases of LmMCF cells in the lungs. Scale bar, 2 mm. **e** Quantification of the ratio of metastatic area to the whole lungs shows MS275 treatment inhibiting lung metastases of LmMCF cells

We next evaluated the effect of the treatments on the in vivo growth of TNBC cells. All XtMCF xenograft tumors in CB17/SCID mice continued growing during the course of therapy; however, the growth of the majority of tumors from treated animals was slower than tumors of control group in the week following the termination of therapy and thereafter. Mice were sacrificed 10 days after treatment stopped, there was a 54% decrease in the median tumor weight in MS275 treated group, and a 52% decrease in SGI + MS275 group (Fig. 5a, b). We also evaluated the effect of the treatments using NOD/SCID mice, with higher doses but a short treatment period. By this schedule, SGI + MS275 treatment reduced tumor weight by 33% compared to control, although SGI or MS275 alone didn’t show much effect on the tumor growth (Additional file 11: Figure S7a-c). Using LmMCF cells and CB17/SCID mice model, all treated groups showed the trend of decrease in tumor weight but it didn’t reach statistical difference (Additional file 11: Figure S7d, e). These results suggest that epigenetic drugs can exert a significant anti-tumor effect on TNBC in vivo when appropriate treatment schedule is used.

The lung metastatic study was conducted using LmMCF cell line, 80,000 cells were injected into the tail vein. MS275 treatment reduced the tumor size on lungs surface compared to control shown by Bouin’s solution fixation (Fig. 5c). The IHC staining to human vimentin on lung section showed the presence of tumor foci in the lungs (Fig. 5d), quantification of the lung areas affected by the metastases showed MS275 reduced lung metastases by 2 fold compared to control (1.9% ± 0.9% for MS275 vs. 3.8% ± 1.5% for control) (Fig. 5e). This result was consistent to the in vitro study that showed MS275 inhibits the migration and invasion of LmMCF cells.

### DNMTi and HDACi up-regulate E-cadherin, induce changes in epithelial cell adhesion molecule (EpCAM), and inhibit WNT signaling

**Fig. 6:**
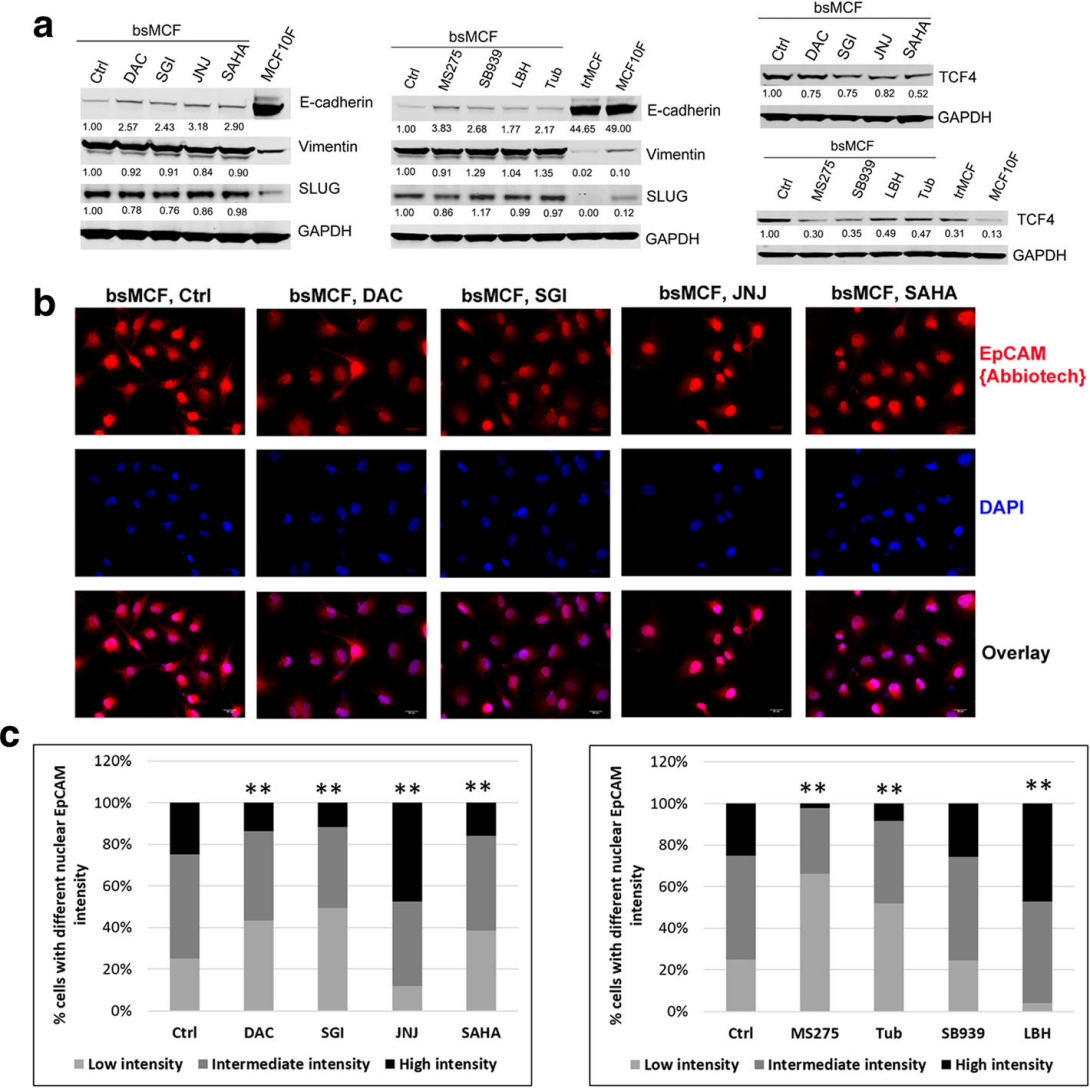
Treatment of DNMTi and HDACi reprogram EMT partially mediated by EpCAM and WNT signaling. **a** Western blotting show up-regulation of E-cadherin and down-regulation of TCF4 by the treatment of DNMTi and HDACi. The number below the band indicates the relative expression level to the control quantified by Ly-Cor Odyssey software. **b** Representative images of immunofluorescence staining of EpCAM. Magnification, 400×. Scale bar, 20 μm. **c** Quantification of nuclear EpCAM shows reduced nuclear EpCAM intensity in the cells treated with DAC, SGI, SAHA, MS275, and Tub, whereas an increased intensity was observed in the cells treated with JNJ and LBH

To investigate the mechanisms underlying the reversal of mesenchymal-like features induced by the treatment of DNMTi and HDACi, Western blot analyses were performed to assess the expression of EMT markers. All treatments induced up-regulation of E-cadherin in bsMCF cells, without significant change of vimentin and SLUG expression (Fig. 6a).

The change of E-cadherin partially explained the reversal of bsMCF mesenchymal phenotype, but it could not address why the treatments of JNJ, SB939, and LBH promotes bsMCF cell invasion while up-regulating E-cadherin. Our previous study suggested that the cleavage of EGF-like domain and the nuclear translocation of EpCAM are associated with EMT process [10]. Therefore, we examined the expression of EpCAM with antibodies targeting different epitopes (Additional file 12: Figure S8a, b). The staining using antibody EpCAM(VU1D9) targeting N-terminal domain showed there was no detectable EpCAM in bsMCF cells. However, using antibody EpCAM{Abbiotech} which recognizes thyroglobulin repeat-like domain and a part of cysteine poor region, we were able to observe the expression of EpCAM in these cells. The quantification of immunofluorescence showed that the nuclear EpCAM intensity was reduced in bsMCF cells treated with DAC, SGI, SAHA, MS275, and Tub, in contrast it was increased in bsMCF cells treated with JNJ and LBH (Fig. 6b, c, and Additional file 13: Figure S9a), suggesting the elevated nuclear EpCAM level may be related to the increased cell invasion in JNJ and LBH treated bsMCF cells. EpCAM nuclear translocation is closely related to the WNT signaling, the intracellular domain of EpCAM can form a nuclear protein complex with beta-catenin, leading to gene transcription [26, 27]. Therefore, TCF4, the main effector of WNT signaling, was examined. All treatments reduced the expression of TCF4, and the change was more pronounced with the treatment of MS275 and SB939 (Fig. 6a). These results suggest both EpCAM and WNT signaling may contribute to the reversal of mesenchymal phenotype of bsMCF cells, but the crosstalk between these two pathways may be affected by the particular drug been used.

### Combined treatment of SGI with MS275 synergistically up-regulates E-cadherin, increases full length EpCAM, and inhibits WNT signaling

**Fig. 7:**
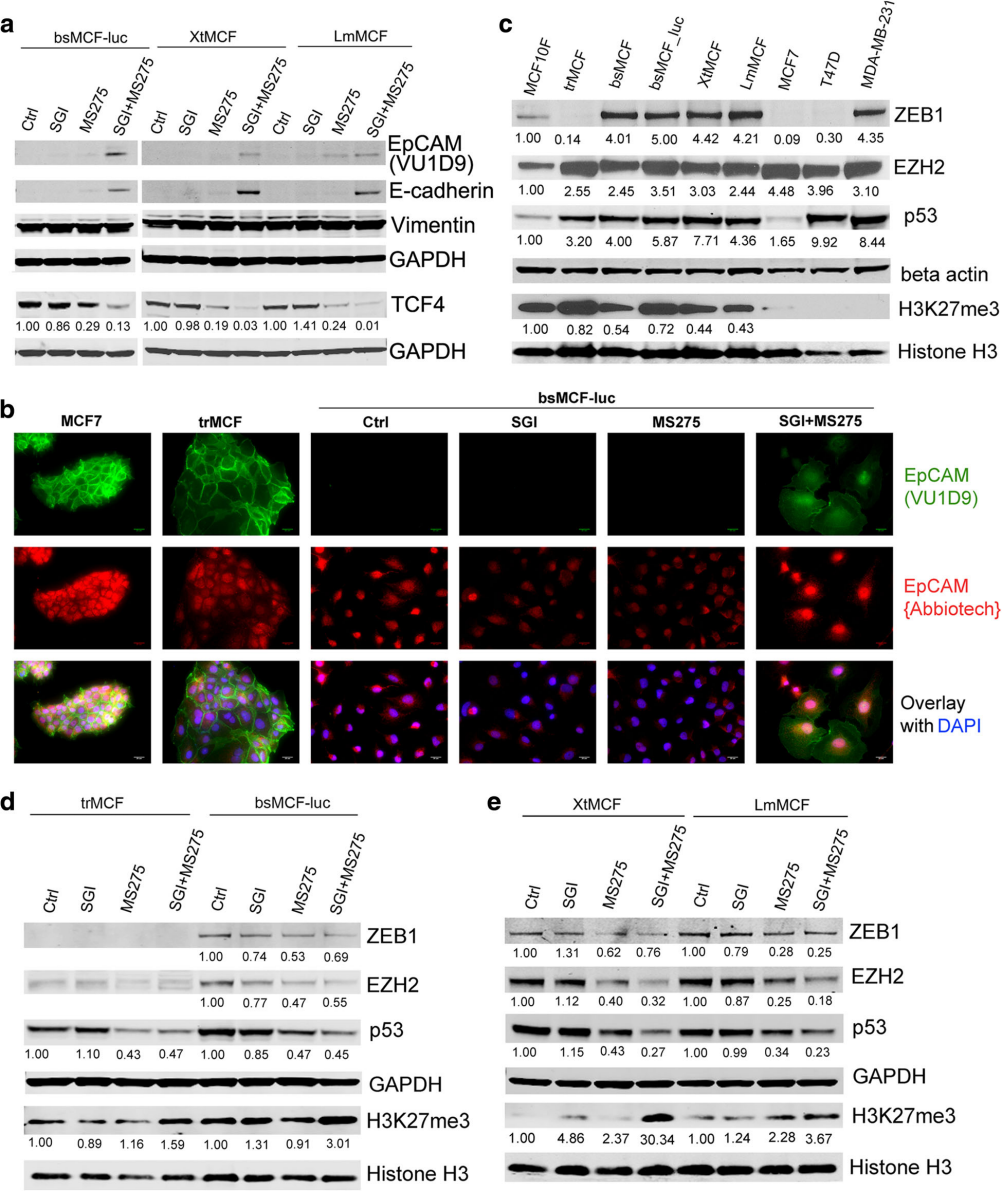
The combination of SGI with MS275 suppresses WNT and mutant p53 pathways, up-regulates H3K27me3. **a** WB analyses show up-regulation of E-cadherin and full length EpCAM, and down-regulation of TCF4 in TNBC cells. **b** Immunofluorescence images show presence of EpCAM containing N-terminal EGF domain indicated by staining with EpCAM(VU1D9) antibody in bsMCF-luc cells after the combined treatment. Magnification, 400×. Scale bar, 20 μm. **c**, **d** and **e** WB analyses of mutant p53, ZEB1, EZH2, and H3K27me3. Beta actin and GAPDH were used as loading control

We next determined whether the combined treatment of SGI with MS275 would be superior to the single agents in regulating these signaling. Figure 7a shows E-cadherin was significantly up-regulated by the combined treatment, whereas it was not detectable in control cells, and was just slightly observed in MS275 treated cells (Fig. 7a). Similarly, the full length EpCAM showed by the reactivity to the antibody EpCAM(VU1D9), was significantly increased in cells treated with the combination (Fig. 7a), and the fluorescence staining further proved the presence of full length EpCAM in these cells (Fig. 7b and Additional file 13: Figure S9b), indicating the combined treatment protects EpCAM from being cleaved. Consistently, TCF4 was significantly reduced in cells treated with the combination when compared with the single agent. These findings suggest that SGI and MS275 have a synergistic effect on up-regulating E-cadherin, and this effect may be partially mediated by inhibiting EpCAM cleavage and WNT signaling.

### Treatment with SGI, MS275, or the combination differentially regulate mutant p53, ZEB1, and EZH2 in TNBC cells

It has been reported that SAHA and MS275 suppress mutant p53 expression in TNBC and pancreatic cancer [28, 29]. *TP53* mutations are the most frequent genetic alterations in breast cancers, especially in basal-like cancers [30]. The mutant p53 can induce ZEB1 and EZH2 expression thus promote EMT [31, 32]. We performed cDNA sequencing of exon 5–8 (central DNA binding domain) of *TP53* gene, and found the tumorigenic cell lines bsMCF, bsMCF-luc, XtMCF, and LmMCF all harbor one copy of mutation in codon 241 (TCC in wild type vs. CCC in mutant), in contrast, the MCF10F and trMCF which present epithelial phenotype do not have this mutation. Study of *TP53* mutation database showed that the mutation in codon 241 is also observed in bladder carcinoma, brain tumor, breast cancer, and other cancer types. Western blotting showed p53 protein was significantly increased in trMCF cells and tumorigenic cell lines. Consistent to the mutation of *TP53*, EZH2 was increased in all transformed cells, and ZEB1 was up-regulated in cells that underwent EMT (Fig. 7c). Treatment of SGI did not change the expression of p53, whereas MS275 treatment significantly reduced p53 in all cell lines. Combined treatment of SGI with MS275 resulted in further suppression in p53 expression in XtMCF and LmMCF cells compared to single agent. Similarly, EZH2 and ZEB1 expression were inhibited by MS275 alone or MS275 combined with SGI (Fig. 7d, e). Our results revealed that MS275, or the combination of MS275 with SGI down-regulates mutant p53 expression and its downstream signaling in TNBC cells.

### Treatment with SGI, MS275, or the combination induce methylation of lysine-27 on histone H3 in TNBC cells

EZH2 is a functional enzymatic component of the polycomb repressive complex2. The primary function of EZH2 is to methylate lysine-27 on histone H3 (H3K27me3). However, EZH2 and H3K27me3 show an inverse correlation across breast cancer subtypes, the EZH2 expression is the highest and the H3K27me3 is the least in TNBC tumors compared to other subtypes [33]. We were interested in determining whether the H3K27me3 level was also altered during the EMT and whether it can be regulated by SGI and MS275 treatment. As shown in Fig. 7c, the global H3K27me3 was lower in mesenchymal-like tumorigenic cells compared to epithelial MCF10F and trMCF cells. SGI or MS275 treatment resulted in an elevated H3K27me3 in XtMCF and LmMCF cells, the combined treatment synergistically increased H3K27me3 in all cell lines, especially in XtMCF (Fig. 7d, e). This result indicates the reversal of EMT may also be mediated by the regulation of epigenetic marker H3K27me3 that is associated with the transcription repression.

### SGI and MS275 have distinct growth inhibitory effect on breast cancer cell lines; combined treatment of SGI with MS275 results in further inhibition

**Fig. 8:**
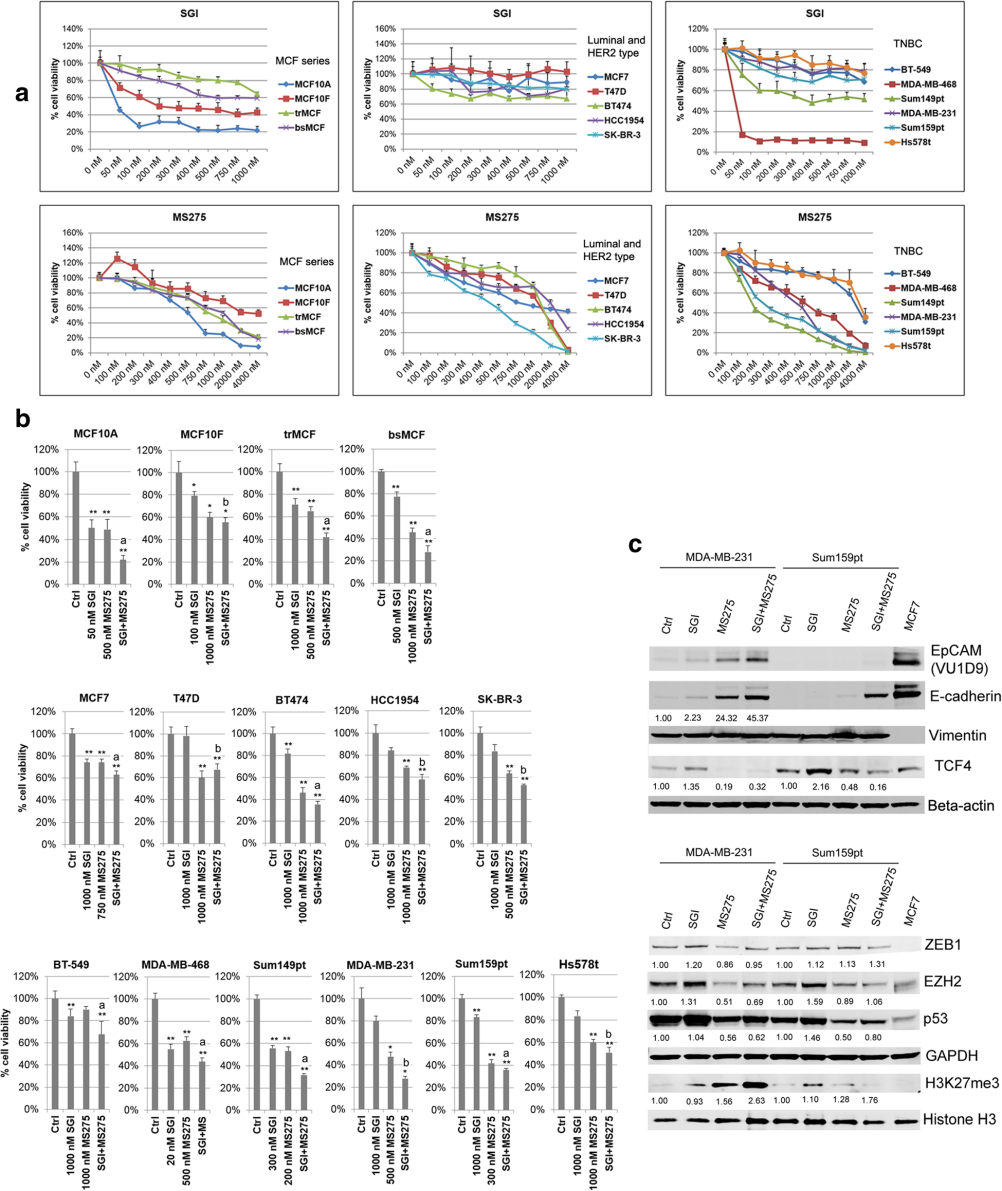
TNBC cell lines are sensitive to the combined treatment of SGI with MS275. **a** Dose response curve of breast epithelial/cancer cell lines to the treatment of SGI and MS275 by MTT assay. **b** The effect of SGI, MS275, or the combination treatment on the growth of breast epithelial/cancer cell lines. **c** Western blotting show inhibition of WNT and mutant p53 signaling pathways, and up-regulation of H3K27me3

To determine whether the growth inhibitory effects of SGI, MS275, and the combination also apply to other breast cancer cell lines, we tested the treatments in a panel of commercial available luminal, HER2 type, and TNBC cell lines. The MCF series cell lines were used for comparison. The growth of luminal and HER2 type cell lines was not sensitive to SGI (Fig. 8a, middle panel). Among the six tested TNBC cell lines (Fig. 8a, right panel), MDA-MB-468 was extremely sensitive to SGI. (Fig. 8a). All tested cell lines showed a dose dependent response upon MS275 treatment. The dose around IC50 was then used to do combined treatment. For the cell lines with the IC50 above 1000 nM, cells were treated with the agent at 1000 nM. Although luminal and HER2 type cell lines did not respond very well to SGI alone at concentrations below 1000 nM, combined treatment showed greater inhibition in cell growth than single agent. The MCF series and all other TNBC cell lines showed significantly inhibitory effect upon the combined treatment, with the most sensitive in MDA-MB-468 and Sum149pt cell lines (Fig. 8b).

To verify our findings that SGI combined with MS275 induced reversal of EMT through different pathways in TNBC cells, two mesenchymal-like cell lines MDA-MB-231 and Sum159pt were used for further studies, Western blotting showed that MS275 significantly induced E-cadherin and full length EpCAM, and reduced TCF4 in MDA-MB-231 cells. Combined treatment induced higher expression of E-cadherin and full length EpCAM than single agent alone in MDA-MB-231. The restoration of full length EpCAM was not observed in Sum159pt cells although the combined treatment showed significant up-regulation of E-cadherin and inhibition of TCF4 (Fig. 8c). Both MDA-MB-231 and Sum159pt cells have *TP53* missense mutation [34]. Western blotting showed there was a notable decrease of p53 in both cell lines after MS275 and the combined treatment. EZH2 was also reduced by MS275 and the combined treatment in MDA-MB-231, but not in Sum159pt cells (Fig. 8c). There was no significant change in ZEB1 expression in both cell lines with any of the treatments. H3K27me3 was increased by MS275, with greater increase by the combined treatment in both cell lines. These results suggest that the growth inhibition and the reversal of EMT by the combined treatment of SGI with MS275 are not limited to our MCF series model, but also apply to other TNBC cells presenting EMT features, nevertheless the involved pathways might be dependent on the cell lines.

## Discussion

The role of EMT in cancer biology is complicated. It is accepted that the EMT plays important roles not only in tumor metastasis, but also in initiation, stemness of cancer cells, and the resistance of cancer cells to treatments. Some transcription factors such as SNAIL1/2, TWIST, and ZEB1/ZEB2 are known key drivers of EMT [35]. Since epigenetic mechanisms are involved in the activation of EMT, in this study, we aimed to investigate the potential of using inhibitors of DNMT and HDAC to treat TNBC by targeting EMT.

Taking advantage of our EMT TNBC cell model [10], we tested three DNMT inhibitors and six HDAC inhibitors, focused on the cell lines that have undergone EMT, and investigated if these epigenetic compounds can remodel EMT process. Here we show that DNMT inhibitors and HDAC inhibitors can reprogram highly aggressive TNBC cells that have undergone EMT to a less aggressive state, as evidenced by reduced cell proliferation, motility, and invasion, as well as colony formation. We found that the second generation DNMTi SGI is superior to the two DNMTi AZA and DAC, and the HDACi MS275 is more potent than other five HDAC inhibitors (SAHA, JNJ, SB939, LBH, and Tub) tested in this study. We demonstrated for the first time that the combined treatment of SGI with MS275 exerts a greater effect than single agent alone in inhibiting cell proliferation, motility, colony formation, and stemness of cancer cells, as well as in inducing apoptosis.

The DNA methylation inhibitors AZA and DAC have been approved for medical use in the treatment of leukemia and myelodysplastic syndrome (MDS), however, the use in breast cancer is still under research [36]. Clinical trial of using DAC before surgery in treating patient with locally advanced HER-2 negative breast cancer is ongoing. SGI, also known as guadecitabine, is a dinucleotide of a decitabine (DAC) linked via phosphodiester bond to a guanosine, this configuration provides protection from drug clearance by deamination, leading to prolonged exposure to decitabine. SGI exerts immunomodulatory action in leukemia [37], ovarian cancer [38], sensitizes cancer cells to other agents [25, 39, 40]. SGI is currently used in clinical trials with non-small lung cancer, recurrent ovarian cancer, metastatic colonrectal cancer, hepatocellular carcinoma, MDS, and leukemia. The investigation of SGI in breast cancer and clinical trial has not been reported yet.

Use of HDAC inhibitors in breast cancer treatment are being extensively studied in recent years. Pan-HDAC inhibitor SAHA was the first HDAC inhibitor approved by the US Food and Drug Administration for the clinical use in 2006. SAHA is currently in phase II clinical trial for patients with hormone receptor positive stage IV breast cancer. Three other pan-HDAC inhibitors, JNJ, LBH, and SB939 are studied for the treatment of different cancers including breast cancer. Tub is a selective HDAC6 inhibitor, it shows a dose-dependent effect on cell viability in MDA-MB-231 and induces apoptosis [41]. MS275 is a selective HDAC1 and HDAC3 inhibitor. MS275 induces apoptosis of erbB2-overexpressing cells [42], sensitizes TRAIL-resistant breast cancer cells, inhibits angiogenesis and metastasis, as well as reverses EMT [43, 44]. MS275 also has an effect in inhibiting tumor-initiating cells [45]. MS275 is currently used in multiple phase III clinical trials in treating breast cancer.

In this study we investigated the mechanism of action of DNMTi and HDACi in TNBC cells, and showed that E-cadherin was significantly induced in mesenchymal-like cancer cells by all treatments, suggesting the reversal of EMT by epigenetic remodeling. E-cadherin is one of the most important molecules in epithelial cells, it interacts with a bundle of proteins and regulates basic cellular process. One protein that E-cadherin interacts with is beta-catenin. Research showed E-cadherin inhibits tumor cell growth by antagonizing beta-catenin signaling [46]. The up-regulation of E-cadherin might contribute to the inhibition of cell growth by DNMTi and HDACi. Furthermore, both DNMTi and HDACi can also inhibit cell proliferation through other anti-tumor effects such as inducing cell cycle arrest [47, 48].

We also found that the expression pattern of EpCAM is closely related to the status of EMT and the invasion property of the cancer cells. The combined treatment of SGI with MS275 significantly increased the full length EpCAM protein, in a pattern that was parallel to the up-regulation of E-cadherin, implying these cell membrane proteins are important to help cells stick to one another (cell adhesion). EpCAM has been considered as an oncogenic signal transducer, the cleaved EpCAM helps relay signals from outside of cells to nucleus, resulting to cross-talk to other signaling including WNT pathway [49]. In our study, we observed a decrease of TCF4 by DNMTi and HDACi in mesenchymal-like cell lines. It is of importance that the combined treatment of SGI with MS275 synergistically increases full-length EpCAM, inhibits TCF4, and upregulates E-cadherin. Based on these findings, we postulate that the cleavage of N-terminal EGF-like domain of EpCAM may cause the internalization of the cleaved EpCAM, which cross-talks with WNT signaling and other pathways, contributing to EMT process. The combined treatment of SGI with MS275 synergistically inhibits the cleavage of EpCAM, thus suppresses WNT signaling and reverses EMT. Giving the fact that WNT/beta-catenin signaling is related to TNBC tumorigenesis and metastasis [50, 51], and there are no drugs targeting WNT pathways currently approved for the treatment of TNBC, our results strongly suggest combined treatment of SGI with MS275 could be a strategy targeting EMT through WNT pathway in TNBC.

In addition, we observed that MS275 alone or combined with SGI suppresses the expression of mutant p53, reduces ZEB1 and EZH2 protein in mesenchymal-like TNBC cells. There is evidence that loss of p53 function is associated with the induction of EMT and acquisition of stemness in different cancer cell lines [52, 53]. ZEB1 is one of the most important E-cadherin repressors in EMT process. ZEB1 can be induced by mutant p53 [31] or loss of wild type p53 [53] through modulating miRNAs. EZH2 is a marker of aggressive breast cancer, overexpression of EZH2 promotes neoplastic transformation of breast epithelial cells [54]. Breast cancers with *TP53* mutation correlate with high EZH2 expression [55]. Jiang et al. demonstrated that mutant p53 induces EZH2 expression and promotes EMT via miR-26a in endometrial carcinoma cells [32]. Thus, the reversal of EMT by MS275 and the combination could be partially through the suppression of mutant p53. Furthermore, both mutant p53 and EZH2 have multiple functions in tumorigenesis other than inducing EMT [56, 57]. SGI combined with MS275 could also be a strategy targeting mutant p53 and EZH2 in TNBC treatment.

One interesting finding in this study is the observation of the significant increase of H3K27me3 level in treated cells. Breast cancer patients with low H3K27me3 have a shorter survival than those with intermediate and high levels [33]. H3K27me3 level is usually maintained by polycomb repressive complex and histone demethylases. KDM6B is a demethylase that specifically demethylates H3K27me3, it is required by TGF beta induced EMT in mammary cells, and is highly expressed in invasive breast cancer [58]. Wang et al. reported that miR-138 suppresses EMT through degradation of KDM6B in breast cancer carcinoma [59]. A more recent study showed that treatment of MCF7 and MDA-MB-231 cells with GSKJ4 inhibits self-renewal of cancer stem cells by inhibiting KDM6B and UTX (another histone demethylase) and elevating the global H3K27me3 level [60]. Based on these studies, we postulate that one of the reasons that causes low H3K27me3 in TNBC could be the over expression of KDM6B, and the combined treatment of SGI with MS275 might probably regulate KDM6B or other histone demethylase through miRNA or other pathways, which result in the increase of H3K27me3 and chromatin remodeling, hence contribute to reversal of EMT.

In this work, although SGI or MS275 treatment alone showed almost similar effect in reversing EMT phenotype in vitro, the in vivo study using CB17/SCID mice showed that MS275 was more potent in suppressing xenograft growth. This could be due to the treatment schedule used for SGI was not optimal. We found that SGI was actually more effective than MS275 in inhibiting colony formation and stemness of TNBC cell lines. Pathania et al. reported that DNMT1 is essential for mammary and cancer stem cell maintenance and tumorigenesis [61]. Assessment of DNMT1 in breast cancer tissues showed that DNMT1 is highly expressed in TNBC compared with other subtype [62]. DNMT1 is a direct target of SGI, treatment of SGI depletes DNMT1 in hepatocellular carcinoma cell line [40]. Moreover, DNMT1 is associated with TGF beta-induced EMT in ovarian cancer cells, and SGI treatment prevents this EMT process [63]. These findings suggest that SGI has the potential to reduce the risk of TNBC metastasis by inhibiting EMT and stemness of cancer cells.

Similar to the other chemotherapies, the use of DNMTi and HDACi in treating solid tumors were not very successful in clinics. Many reasons such as toxicity, poor penetration, drug resistance, and poor patient section could attribute to this failure. Compared to other DNMTi, SGI-110 has a prolonged half-life that allows to use it at a lower dose and longer interval thus reducing the toxicity. MS275 is a selective HDAC1 and HDAC3 inhibitor, it has multiple anti-tumor effects. In the present study, we focus on EMT only and observed that SGI combined with MS275 reverses EMT in mesenchymal-like TNBC cells. We showed that TNBC cell lines are more sensitive to the treatment of SGI combined with MS275 compared with other subtypes, implying the clinical significance in patient selection. Taken together, our results clearly suggest that the combination of SGI with MS275 may have clinical advantage over other DNMTi and HDACi in treating TNBC.

## Conclusions

Our study showed that DNMTi and HDACi exert antitumor activity in TNBC cells partially by epigenetically reprograming EMT. We demonstrated that the DNMTi SGI and HDACi MS275 have synergistic effect on reversing EMT by inhibiting EpCAM cleavage and WNT signaling, suppressing mutant p53, ZEB1, and EZH2 expression, and inducing E-cadherin, apoptosis, as well as histone H3 lysine 27 tri-methylation. Our findings strongly suggest that TNBC is sensitive to epigenetic based therapies. We propose a new strategy to treat TNBC by using the combination of SGI-110 with MS275, which exerts superior antitumor effects by simultaneously targeting multiple pathways.

## Additional files

Additional file 1: Supplementary methods. (DOCX 24 kb)

Additional file 2: **Table S1.** Subtypes of breast epithelial/cancer cell lines. (DOCX 22 kb)

Additional file 3: **Table S2.** Drug information. (DOCX 22 kb)

Additional file 4: **Table S3.** Antibody information (DOCX 24 kb)

Additional file 5: **Figure S1.** Effects of DNMTi and HDACi on cell proliferation of triple negative breast epithelial/cancer cell lines. (TIF 453 kb)

Additional file 6: **Figure S2.** Effects of HDACi on the growth of bsMCF cells in collagen or agar methylcellulose. (TIF 3904 kb)

Additional file 7: **Figure S3.** Effects of SGI, MS275, and the combination on cell migration and invasion of bsMCF cells. (TIF 1033 kb)

Additional file 8: **Figure S4.** SGI and MS275 inhibit cell proliferation of bsMCF_luc, XtMCF, and LmMCF. (TIF 1491 kb)

Additional file 9: **Figure S5.** Effect of SGI, MS275, or the combination on colony formation. (TIF 696 kb)

Additional file 10: **Figure S6.** Effects of SGI, MS275, and SGI + MS275 on cell migration and invasion of xtMCF and LmMCF. (TIF 1890 kb)

Additional file 11: **Figure S7.** Treatment of SGI, MS275, or the combination in xenograft model. (TIF 734 kb)

Additional file 12: **Figure S8.** N-terminal EGF-like domain of EpCAM is cleaved off after cells underwent EMT. (TIF 1507 kb)

Additional file 13: **Figure S9.** Immunofluorescence staining of cells treated with single or combined agent. (TIF 1960 kb)

## Abbreviations

AZA: 5-Azacytidine
DAC: Decitabine
DMSO: Dimethyl sulfoxide
DNMTi: DNA methyltransferase inhibitors
EMT: Epithelial mesenchymal transition
FCCC: Fox chase cancer center
HDACi: Histone deacetylase inhibitors
i.p: Intraperitoneal
IF: Immunofluorescence
JNJ: JNJ-26481585
MS275: Entinostat
s.c: Subcutaneous
SAHA: Vorinostat
SGI: SGI-110 (Guadecitabine)
TNBC: Triple negative breast cancer
Tub: Tubastatin A HCL
WB: Western blotting
