# Supplementary material for: Epigenetic reprogramming of epithelial mesenchymal transition in triple negative breast cancer cells with DNA methyltransferase and histone deacetylase inhibitors
Source: J Exp Clin Cancer Res. 2018 Dec 14;37:314. doi: 10.1186/s13046-018-0988-8 (PMC6295063; doi:10.1186/s13046-018-0988-8)
Supplement: Supplementary file 1 — Supplementary methods. (DOCX 24 kb) [file 13046_2018_988_MOESM1_ESM.docx]

**Additional file 1: Supplementary methods**

**Cell culture medium**

Triple negative human breast epithelial/cancer cell line MCF10A, MCF10F, trMCF, and bsMCF were cultured in Dulbecco’s modified Eagle medium (DMEM): F12 containing 1.05 mM Calcium, antibiotics, hormones, growth factors and 5% horse serum. T47D, HCC1954, BT-549, MDA-MB-231, MDA-MB-468, and Hs578t were maintained in RPMI-1640 with 10% FBS. MCF7 was maintained in DMEM with 10% FBS. SK-BR-3 was cultured in McCoy’s 5a media with 10% FBS. Sum149pt and Sum159pt were cultured in Ham’s F12 media with 10% FBS, 1 µg/ml hydrocortisone, 50 µg/ml insulin. All cells were maintained in a 37ºC incubator supplied with 5% CO_2_. BT474 cells were cultured in DMEM/F12 with 10%FBS, 2mM L-glutamine, 0.14 units/ml insulin. MCF7, T47D, BT474, SK-BR-3, BT-549, MDA-MB-468, MDA-MB-231, and Hs578t were from cell culture facility at Fox Chase Cancer Center (FCCC). HCC1954 was from American Type Culture Collection (ATCC). Sum149pt and Sum159pt were from Asterand (Detroit, MI).

**MTT cell proliferation assay**

Cells were plated in 96-well plates at a density of 500 to 3000 cells/well on day 1, allowed to attach overnight, and then treated with drug or vehicle in fresh media on day 2. For the combined treatment, cells were incubated with fresh media containing both agents on day 2. Cell proliferation was measured on day 6 (96 hours after treatment) by using Vybrant MTT (3-(4,5-dimethylthiazolyl-2)-2,5-diphenyltetrazolium bromide) Cell Proliferation Assay Kit (Molecular Probes, Eugene, OR). IC50 was calculated using ED50plus v1.0 software.

**Matrigel invasion assay**

Invasion assay was performed using 24-well BioCoat Matrigel invasion chambers (BD Biosciences, Bedford, MA). 750 µl of media containing 20% horse serum was added to the bottom well as chemoattractant. Drug-treated or control cells were seeded in four replicates in the insert at a concentration of 25,000 cells per insert in 500 ul media and incubated for 24 hours. The invasive cells on the lower surface were stained with Giemsa stain, photographed and counted. Data was presented as percent of control which divided cell number of treated group by that of control.

**RTCA migration and invasion assay**

bsMCF, XtMCF, or LmMCF cells were treated with SGI-110, MS275, or the combination, at the dose around IC50 (refers to high dose) or a dose that inhibits cell growth around 20% ( refers to low dose). 96 hours after treatment, cells were trypsinized, and the viable cells were seeded in RTCA plate in medium containing 5% horse serum to avoid the stress caused by serum starving. For bsMCF and XtMCF cells, medium containing 20% horse serum was used as a chemoattractant. For LmMCF cells, 100 ng/ml FGF basic (Sigma, St. Lois, MO) was added to the medium containing 20% horse serum as a chemoattractant. Cell index values were detected every 15 minutes throughout the procedure.

**Immunofluorescence (IF)**

Cells were cultured and treated on 4-well chamber slides (Millipore, Burlington, MA). At the end of treatment, media was removed and the cells were washed with PBS, followed with fixation in 10% buffered formalin, permeabilized. Cells were stained with antibodies vimentin, EpCAM{Abbiotech}, EpCAM(VU1D9), or EpCAM(E144). For the detection of cell apoptosis, cells were plated in plastic flasks, treated with the drug or vehicle on the following day and cultured for 96 hours, then the adherent cells were trypsinized, combined with the floated cells. Cell viability was examined by trypan blue staining, and then 100,000 cells were cytospun on glass slides, fixed with formalin, and stained with cleaved caspase 3. The expression of proteins was detected using Alex Fluor^®^ 488 goat anti-mouse antibody or Alex Fluo^®^ 555 goat anti-rabbit antibody. Nuclei were counterstained with DAPI (Thermo Fisher Scientific). Fluorescent images were captured using Olympus BX53 fluorescent microscope with RetigaTM 2000R Fast 1934 Digital CCD Camera-Monochrome (QIMAGING Corporation, Burnaby, BC, Canada) and MetaMorph software. The fluorescent intensity was quantified using MetaMorph version 7.7.8.0 (Molecular Devices, Sunnyvale, CA).

**Western blotting (WB)**

Total cell lysates were prepared using radio immune precipitation assay (RIPA) buffer (Cell Signaling Technology, Inc. MA) supplemented with protease inhibitors and phosphatase inhibitors. Forty µg proteins were separated on 4%-12% Bis-Tris NuPAGE gels (Life Technologies, Carlsbad, CA) and transferred to nitrocellulose membranes. After blocking with Odyssey blocking buffer, the membranes were incubated with primary antibodies overnight at 4°C. Beta actin or GAPDH was used as loading control. The blots were detected using Li-Cor Odyssey imaging system (Li-Cor Biotechnologies Corporation, Lincoln, NE) or ECL^TM^ Western Blotting Reagents (SIGMA, St. Louis, MO).
