# Supplementary material for: Epigenetic reprogramming of epithelial mesenchymal transition in triple negative breast cancer cells with DNA methyltransferase and histone deacetylase inhibitors
Source: J Exp Clin Cancer Res. 2018 Dec 14;37:314. doi: 10.1186/s13046-018-0988-8 (PMC6295063; doi:10.1186/s13046-018-0988-8)
Supplement: Supplementary file 2 — Table S1. Subtypes of breast epithelial/cancer cell lines. (DOCX 22 kb) [file 13046_2018_988_MOESM2_ESM.docx]

**Additional file 2: Table S1. Subtypes of breast epithelial/cancer cell lines**

| **Cell line** | **Subtype** |
| --- | --- |
| **MCF10A** | **Triple negative breast epithelial cell line** |
| **MCF10F** | **Triple negative breast epithelial cell line** |
| **trMCF** | **Transformed triple negative breast epithelial cell line** |
| **bsMCF** | **TNBC, basal-B subtype** |
| **bsMCF-luc** | **TNBC, basal-B subtype** |
| **XtMCF** | **TNBC, basal-B subtype** |
| **LmMCF** | **TNBC, basal-B subtype** |
| **MCF7** | **Luminal A cell line** |
| **T47D** | **Luminal A cell line** |
| **BT474** | **Luminal B cell line** |
| **SK-BR-3** | **HER2 type** |
| **HCC1954** | **HER2 type** |
| **MDA-MB-468** | **TNBC, basal-A subtype** |
| **Sum149pt** | **TNBC, basal-A subtype** |
| **Hs578t** | **TNBC, basal-B subtype** |
| **BT-549** | **TNBC, basal-B subtype** |
| **MDA-MB-231** | **TNBC, basal-B subtype** |
| **Sum159pt** | **TNBC, basal-B subtype** |
