# Supplementary material for: Epigenetic reprogramming of epithelial mesenchymal transition in triple negative breast cancer cells with DNA methyltransferase and histone deacetylase inhibitors
Source: J Exp Clin Cancer Res. 2018 Dec 14;37:314. doi: 10.1186/s13046-018-0988-8 (PMC6295063; doi:10.1186/s13046-018-0988-8)
Supplement: Supplementary file 3 — Table S2. Drug information. (DOCX 22 kb) [file 13046_2018_988_MOESM3_ESM.docx]

**Additional file 3: Table S2. Drug information**

| Drug name (Abbreviation) | Provider (Catalogue) | Solvent | Stock solution | Drug category |
| --- | --- | --- | --- | --- |
| 5-Azacytidine (AZA) | Sigma (A2385) | DMSO | 100 mM | DNMT inhibitor |
| 5-Aza-2’-deoxycytindine (DAC) | Sigma (A3656) | DMSO | 10 mM | DNMT inhibitor |
| SGI-110 sodium salt (SGI) | Astex (CK1463) | Distilled water | 20 mM | DNMT inhibitor |
| SGI-110 powder for injection (SGI) | Astex | DMSO | 10 mM | DNMT inhibitor |
| JNJ-26481585 (JNJ) | Selleckchem (S1096) | DMSO | 10 mM | HDAC inhibitor |
| Vorinostat (SAHA) | Selleckchem (S1047) | DMSO | 20 mM | HDAC inhibitor |
| Entinostat (MS275) | Selleckchem (S1053) | DMSO | 20 mM | HDAC inhibitor |
| SB939 | Selleckchem (S1515) | DMSO | 10 mM | HDAC inhibitor |
| LBH589 (LBH) | Selleckchem (S1030) | DMSO | 20 mM | HDAC inhibitor |
| Tubastatin A HCL (Tub) | Selleckchem (S2627) | DMSO | 20 mM | HDAC inhibitor |
