# Supplementary material for: Epigenetic reprogramming of epithelial mesenchymal transition in triple negative breast cancer cells with DNA methyltransferase and histone deacetylase inhibitors
Source: J Exp Clin Cancer Res. 2018 Dec 14;37:314. doi: 10.1186/s13046-018-0988-8 (PMC6295063; doi:10.1186/s13046-018-0988-8)
Supplement: Supplementary file 4 — Table S3. Antibody information (DOCX 24 kb) [file 13046_2018_988_MOESM4_ESM.docx]

Additional file 4: Table 3. Antibody information

|  | Antibody name(clone) | Provider | Catalog# | Dilution (IF) | Dilution  (WB) | Dilution  (IHC) |
| --- | --- | --- | --- | --- | --- | --- |
| 1 | E-cadherin(36) | BD Biosciences | 610182 |  | 1:1000 |  |
| 2 | vimentin(V9) | DAKO | M0725 | 1:200 | 1:1000 |  |
| 3 | vimentin(V9) | BioGenex | AM074-5M |  |  | R-T-U |
| 4 | Cleaved caspase 3 | Cell signaling | 9661S | 1:200 |  |  |
| 5 | EpCAM(VU1D9) | Cell Signaling | 2929 | 1:400 | 1:1000 |  |
| 6 | EpCAM{Abbiotech} | Abbiotech | 251617 | 1:200 |  |  |
| 7 | EpCAM(E144) | Abcam | ab32392 | 1:200 | 1:400 |  |
| 8 | TCF4(EP2033Y) | EMD Millipore | 04-1080 |  | 1:1000 |  |
| 9 | p53 | Santa Cruz | SC126 |  | 1:1000 |  |
| 10 | EZH2 | BD Biosciences | 612666 |  | 1:500 |  |
| 11 | ZEB1 | Cell Signaling | 3396S |  | 1:1000 |  |
| 12 | Beta actin | BioVision | 3598-100 |  | 1:2000 |  |
| 13 | GAPDH | Cell Signaling | 5174S | 1:2000 |  |  |
| 14 | H3K27me3 | Cell Signaling | 9733S |  | 1:1000 |  |
| 15 | Histone H3 | Cell Signaling | 14269 |  | 1:1000 |  |
| 16 | Anti-mouse IgG, Alex Fluor^TM^ 488 | Cell Signaling | 4408S | 1:400 |  |  |
| 17 | Anti-rabbit IgG, Alex Fluor^TM^ 488 | Cell Signaling | 4413S | 1:400 |  |  |

R-T-U: Ready to use
