# Supplementary figures and images for: Epigenetic reprogramming of epithelial mesenchymal transition in triple negative breast cancer cells with DNA methyltransferase and histone deacetylase inhibitors
Source: J Exp Clin Cancer Res. 2018 Dec 14;37:314. doi: 10.1186/s13046-018-0988-8 (PMC6295063; doi:10.1186/s13046-018-0988-8)

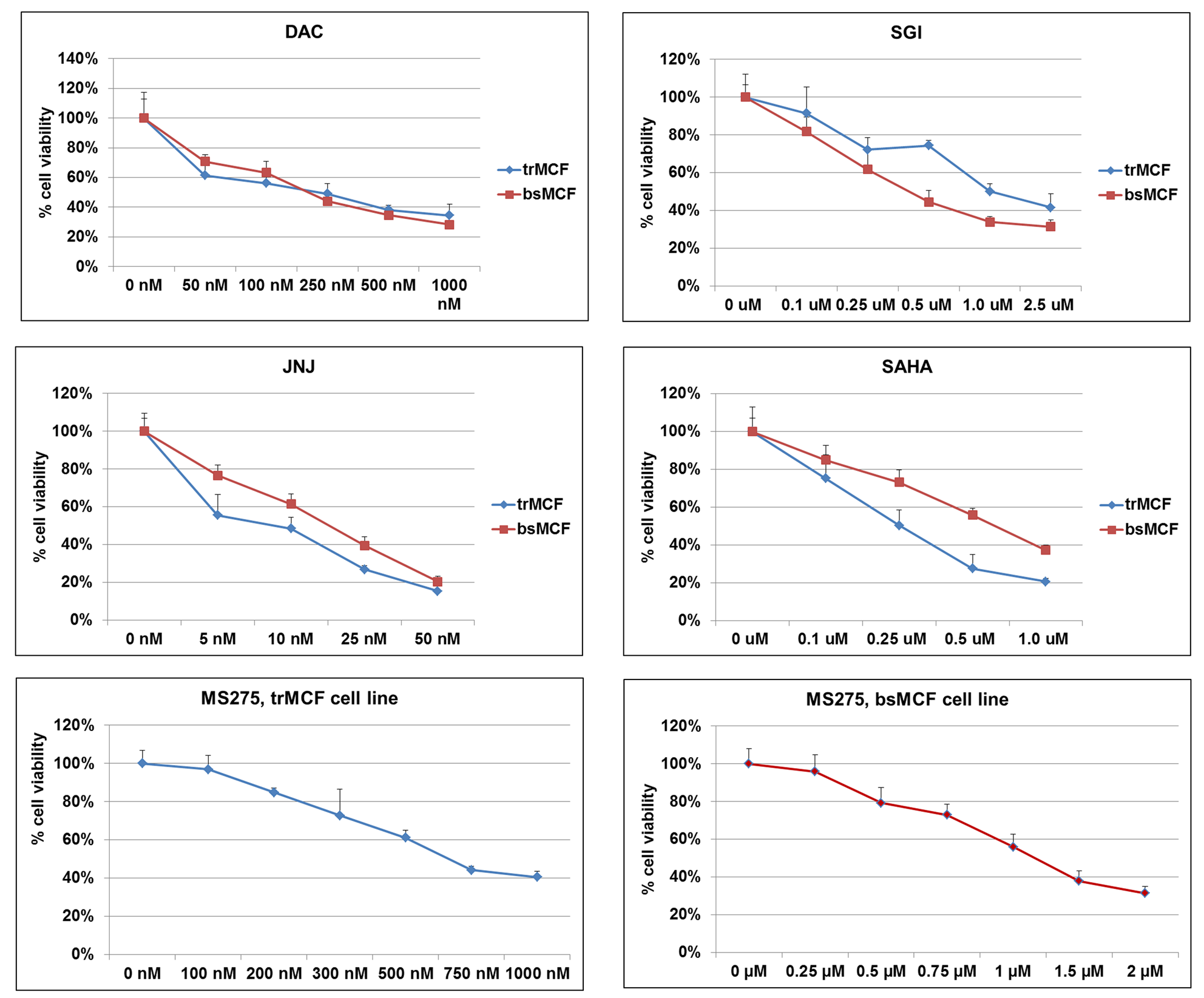

Supplement: Supplementary file 5 — Figure S1. Effects of DNMTi and HDACi on cell proliferation of triple negative breast epithelial/cancer cell lines. (TIF 453 kb) [file 13046_2018_988_MOESM5_ESM.tif]

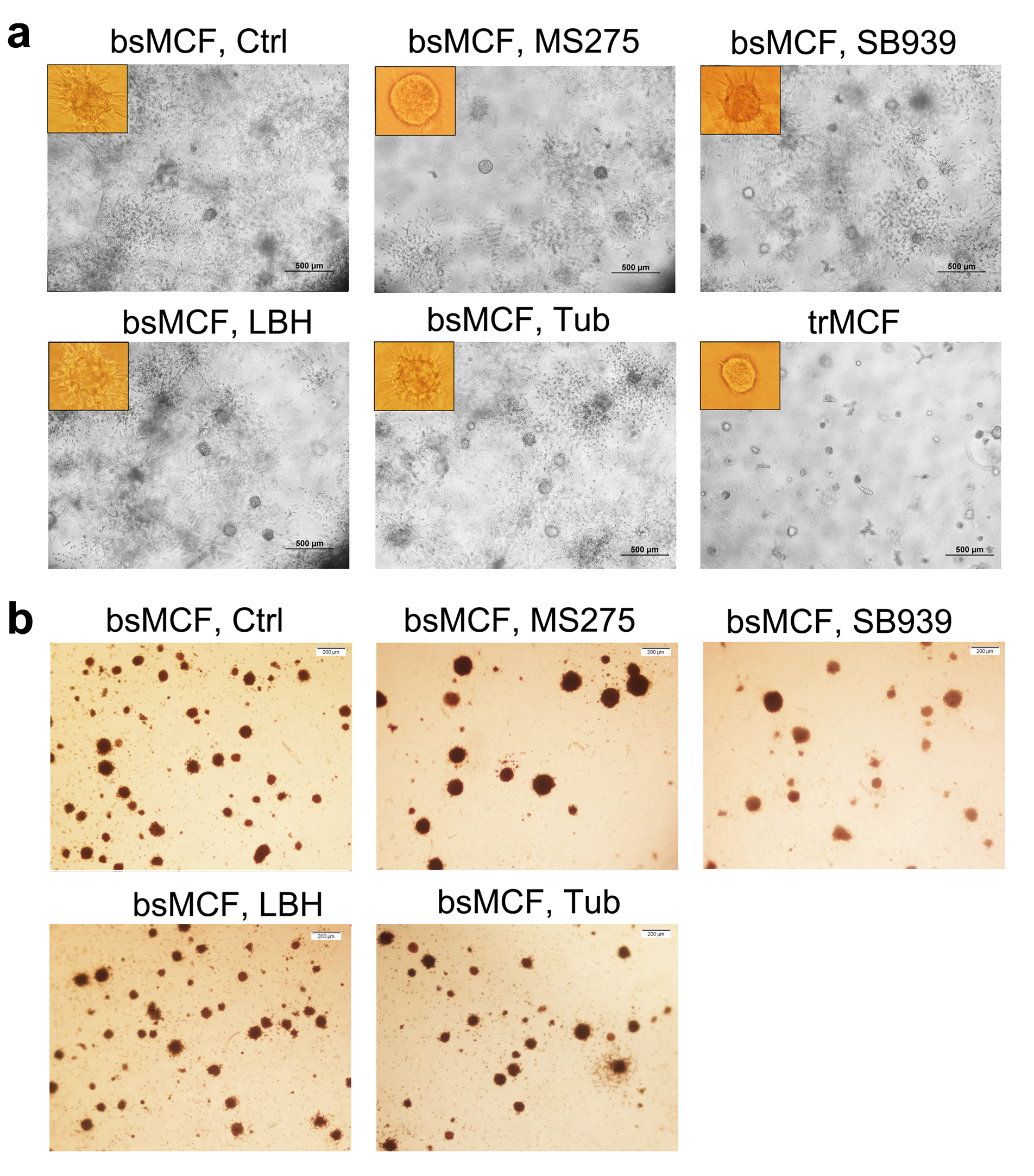

Supplement: Supplementary file 6 — Figure S2. Effects of HDACi on the growth of bsMCF cells in collagen or agar methylcellulose. (TIF 3904 kb) [file 13046_2018_988_MOESM6_ESM.tif]

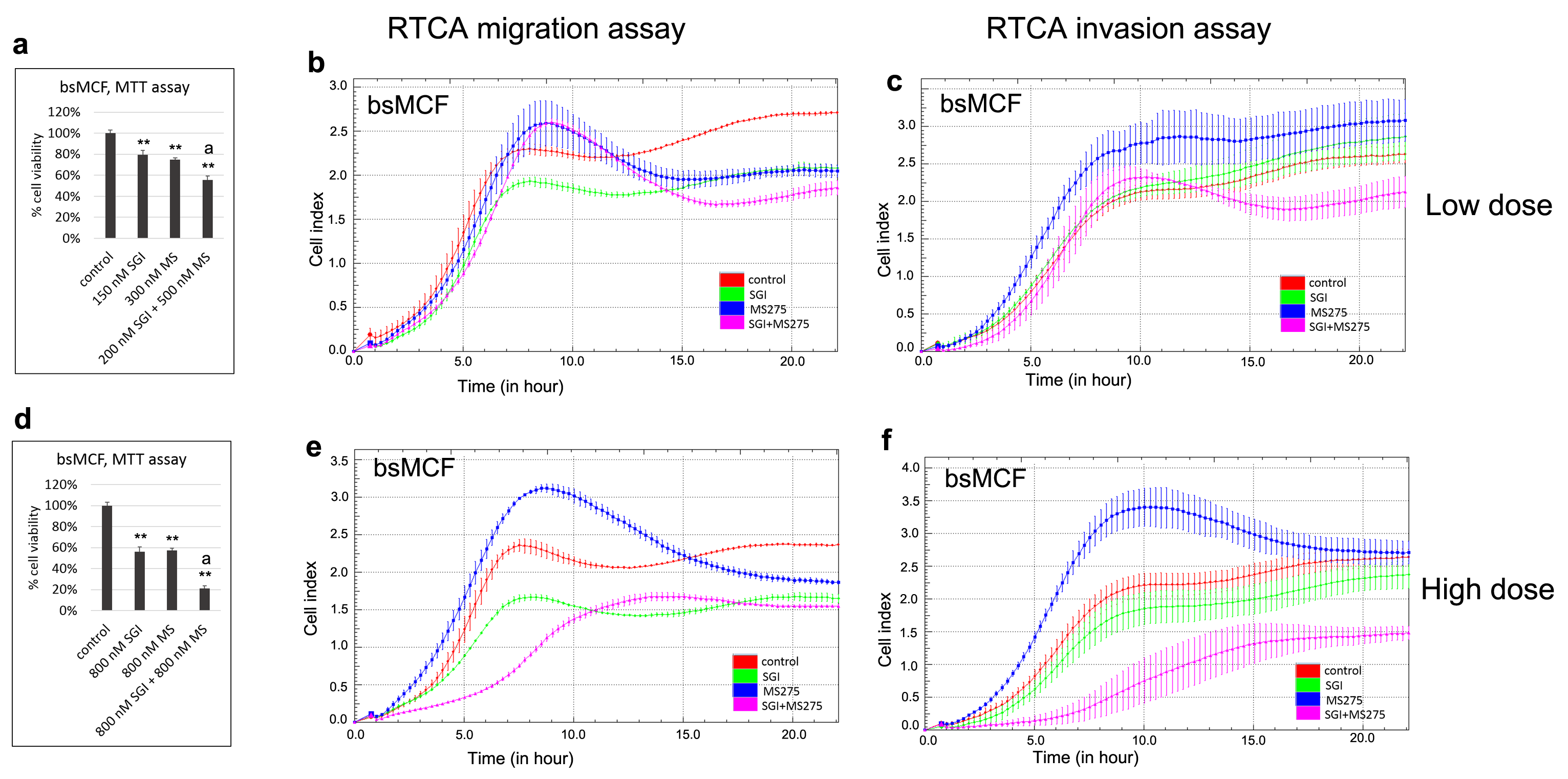

Supplement: Supplementary file 7 — Figure S3. Effects of SGI, MS275, and the combination on cell migration and invasion of bsMCF cells. (TIF 1033 kb) [file 13046_2018_988_MOESM7_ESM.tif]

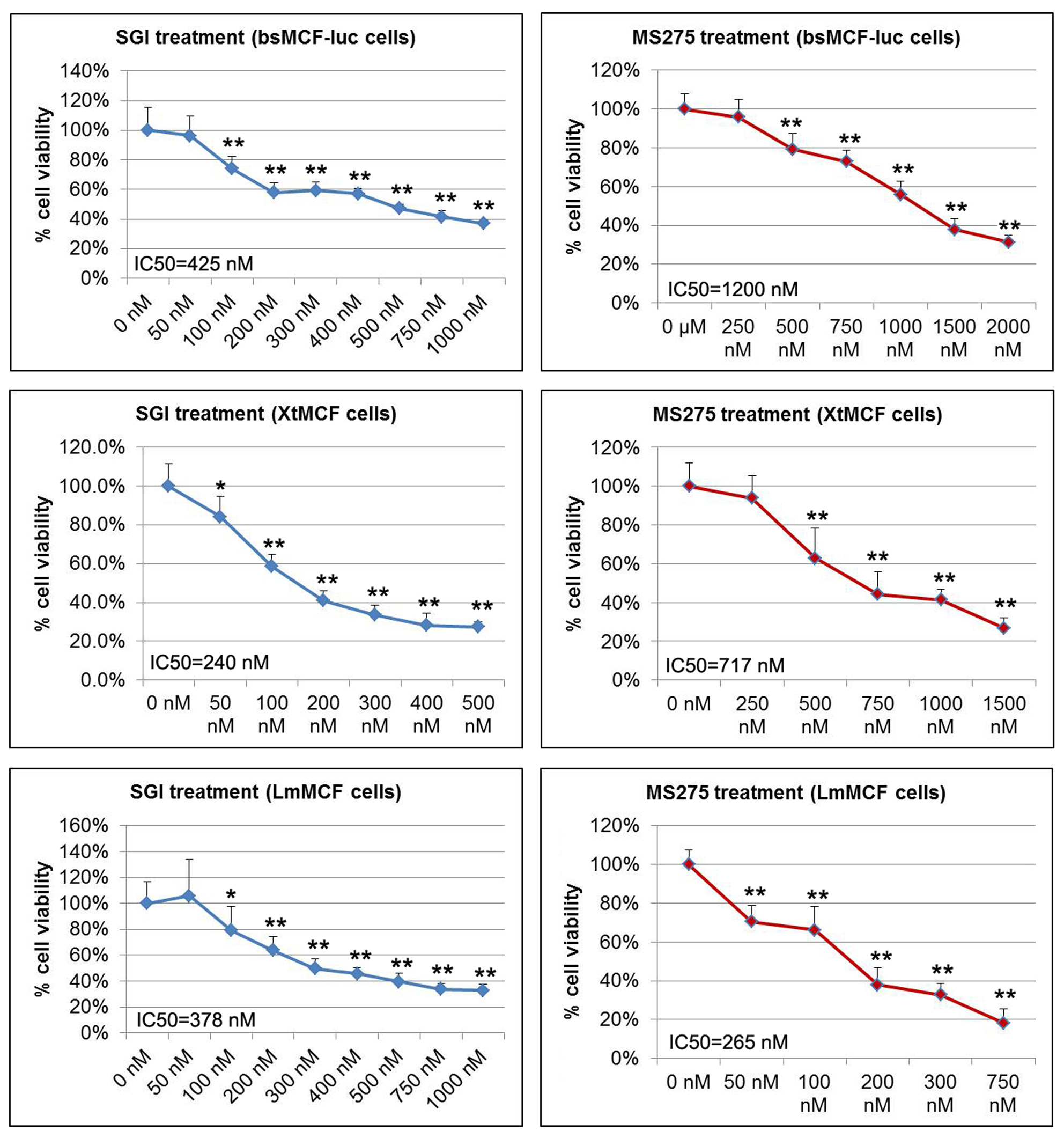

Supplement: Supplementary file 8 — Figure S4. SGI and MS275 inhibit cell proliferation of bsMCF_luc, XtMCF, and LmMCF. (TIF 1491 kb) [file 13046_2018_988_MOESM8_ESM.tif]

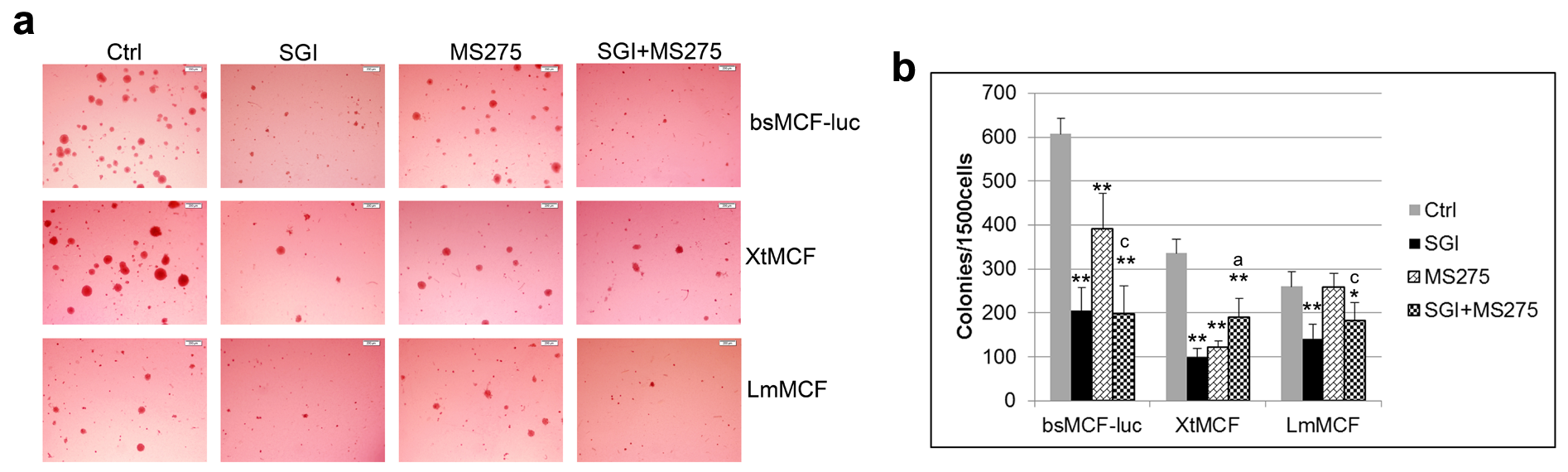

Supplement: Supplementary file 9 — Figure S5. Effect of SGI, MS275, or the combination on colony formation. (TIF 696 kb) [file 13046_2018_988_MOESM9_ESM.tif]

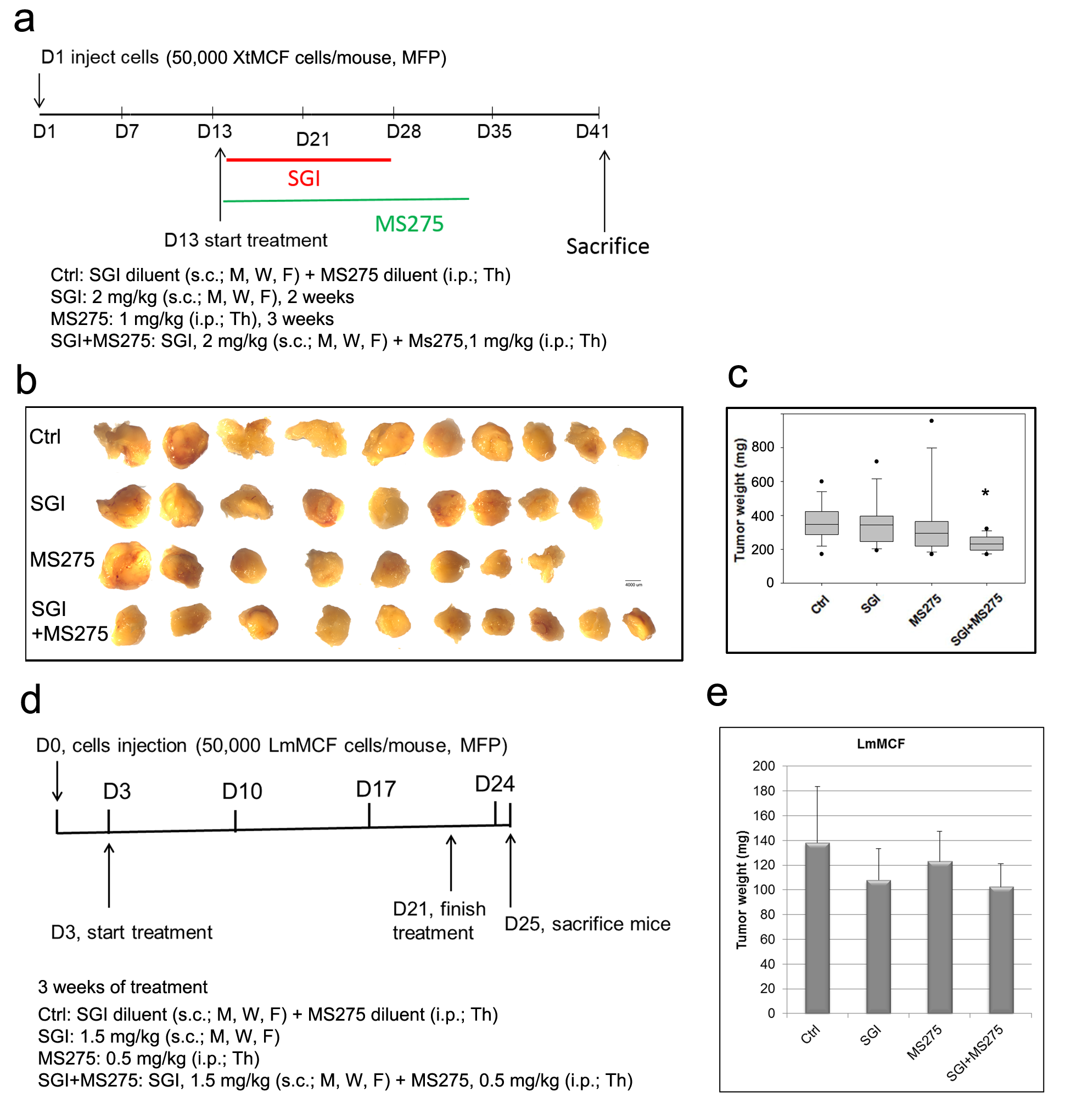

Supplement: Supplementary file 11 — Figure S7. Treatment of SGI, MS275, or the combination in xenograft model. (TIF 734 kb) [file 13046_2018_988_MOESM11_ESM.tif]

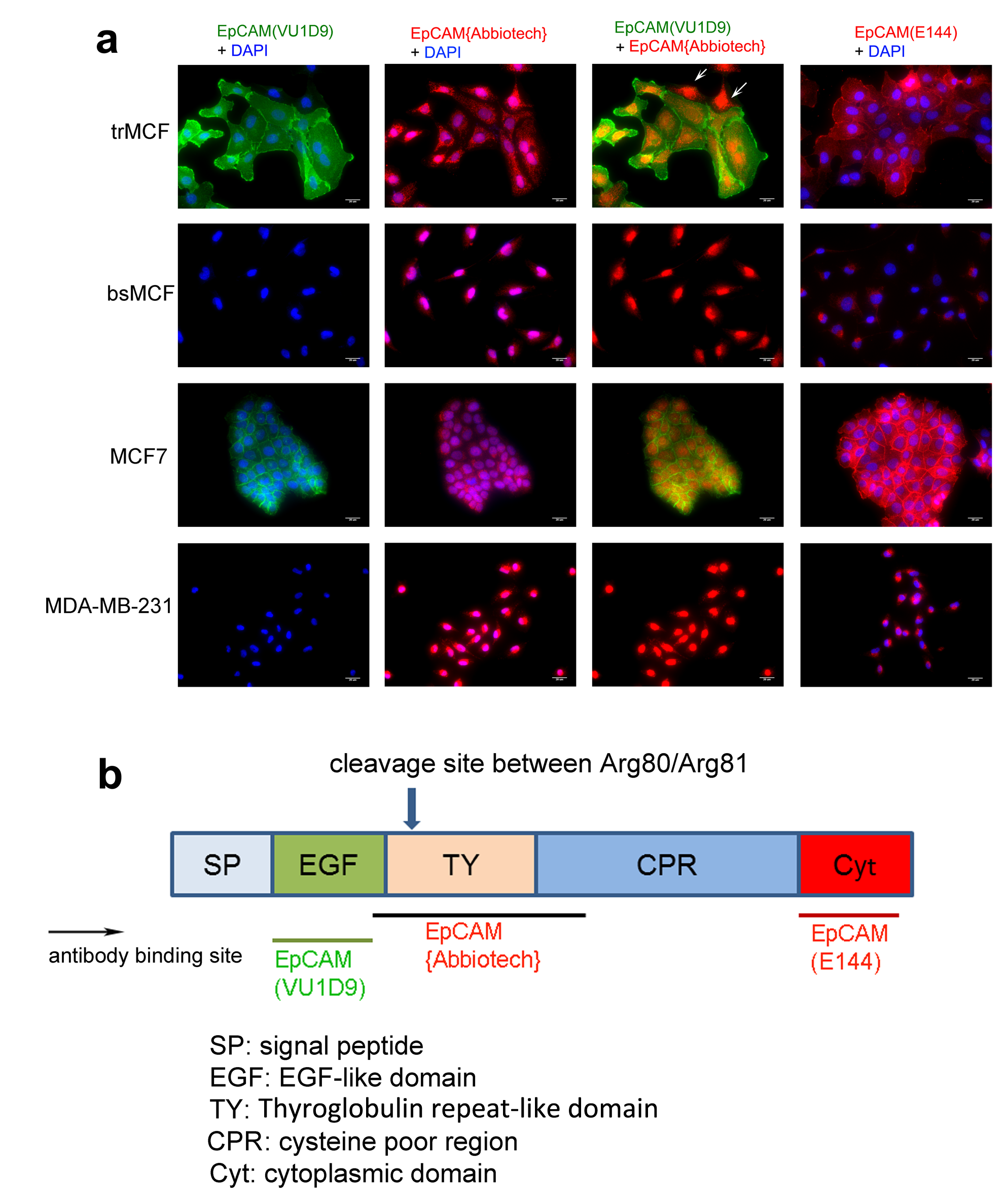

Supplement: Supplementary file 12 — Figure S8. N-terminal EGF-like domain of EpCAM is cleaved off after cells underwent EMT. (TIF 1507 kb) [file 13046_2018_988_MOESM12_ESM.tif]

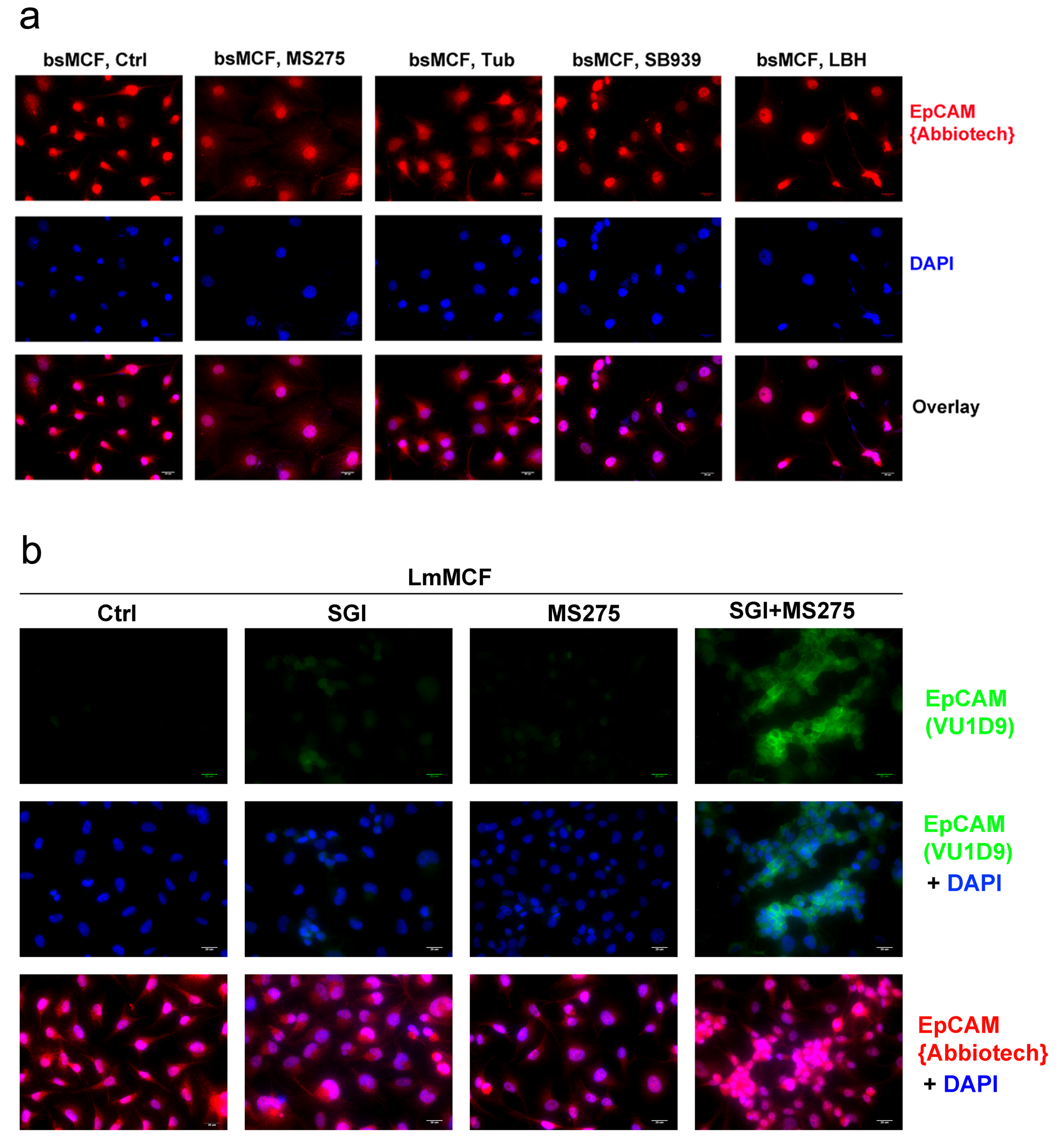

Supplement: Supplementary file 13 — Figure S9. Immunofluorescence staining of cells treated with single or combined agent. (TIF 1960 kb) [file 13046_2018_988_MOESM13_ESM.tif]
